# The c-Myc/miR-27b-3p/ATG10 regulatory axis regulates chemoresistance in colorectal cancer

**DOI:** 10.7150/thno.37621

**Published:** 2020-01-12

**Authors:** Wu Sun, Jialu Li, Likun Zhou, Jiayi Han, Rui Liu, Haiyang Zhang, Tao Ning, Zhiying Gao, Baorui Liu, Xi Chen, Yi Ba

**Affiliations:** 1 Tianjin Medical University Cancer Institute and Hospital, National Clinical Research Center for Cancer, Key Laboratory of Cancer Prevention and Therapy, Tianjin's Clinical Research Center for Cancer, Tianjin, China.; 2 State Key Laboratory for Oncogenes and Related Genes, Key Laboratory of Gastroenterology and Hepatology, Ministry of Health, Division of Gastroenterology and Hepatology, Shanghai Institute of Digestive Disease, Renji Hospital, School of Medicine, Shanghai Jiao Tong University, Shanghai, China.; 3 State Key Laboratory of Pharmaceutical Biotechnology, Jiangsu Engineering Research Center for MicroRNA Biology and Biotechnology, NJU Advanced Institute for Life Sciences (NAILS), School of Life Sciences, Nanjing University, Nanjing, China.; 4 The Comprehensive Cancer Centre of Drum Tower Hospital, Medical School of Nanjing University and Clinical Cancer Institute of Nanjing University, Nanjing, China.

**Keywords:** miR-27b-3p, ATG10, chemoresistance, colorectal cancer, autophagy

## Abstract

Oxaliplatin (OXA) resistance is the major obstacle to the anticancer effects of chemotherapy in colorectal cancer (CRC) patients. MicroRNAs (miRNAs) play an important role in the chemoresistance of various tumors. Our objective is to clarify the underlying mechanism of miRNAs in chemoresistance and provide a potential strategy to improve the response of CRC patients to chemotherapeutics.

**Methods**: MiRNA microarray and Real-time PCR were performed to compare changes in miRNA expression between oxaliplatin-resistant and the parental cells. CCK8, apoptosis assay, immunofluorescence and xenograft studies were used to elucidate the impact of miR-27b-3p on regulating chemoresistance. Luciferase reporter assay and western blot were carried to assess the regulatory role of miR-27b-3p in ATG10 expression. The effects of miR-27b-3p and ATG10 on autophagy were investigated by GFP-LC3 fluorescence microscopy, transmission electron microscopy, and western blot. ChIP assay and luciferase assay were performed to test the c-Myc's occupancy on the miR-27B promoter.

**Results**: We observed that miR-27b-3p expression was significantly downregulated in oxaliplatin-resistant cell lines (SW480-OxR and HCT116-OxR) compared to the corresponding parental cell lines and that miR-27b-3p expression was positively correlated with disease-free survival (DFS) time in colorectal cancer patients. MiR-27b-3p could sensitize colorectal cancer cells to oxaliplatin in vitro and in vivo. Under oxaliplatin treatment, chemoresistant cells showed a higher autophagy level than parental cells. Moreover, we also identified that miR-27b-3p inhibited the expression of ATG10 at the posttranscriptional level, thus inhibiting autophagy. Further study demonstrated that c-Myc can inhibit the expression of miR-27b-3p via binding to the promoter region of miR-27B gene.

**Conclusions**: Our study identifies a novel c-Myc/miR-27b-3p/ATG10 signaling pathway that regulates colorectal cancer chemoresistance. These results suggest that miR-27b-3p is not only a potential indicator for evaluating efficiency of chemotherapy, but also a valuable therapeutic target for CRC, especially for patients with chemoresistance.

## Introduction

Colorectal cancer (CRC) has one of the highest incidence rates among malignant neoplasia and is the main cause of cancer deaths worldwide 1. According to statistics, over 1.8 million new cases of colorectal cancer and 881,000 deaths from this disease occurred in 2018 2. Metastasis is present at diagnosis in 1/4 of the cases, and another 1/4 of CRC patients will subsequently develop metastases within 5 years 3. As a component of first- and second-line combination therapies, oxaliplatin is used to treat metastatic colorectal cancer (mCRC) and has significantly improved response rates to greater than 50% and led to a significant increase in median survival times 4,5. However, the majority of CRC patients will eventually develop drug resistance, and the five-year survival rate for advanced CRC patients is lower than 10% 6. Thus, it is important to illuminate the mechanism of chemoresistance because this knowledge may develop new strategies to overcome drug resistance in CRC patients.

MiRNAs are small noncoding RNAs that control genes expression at the posttranscriptional level 7. As a vital regulator of numerous cell biological processes, numerous miRNAs have been shown to be involved in tumor progression and response to therapy 8. Evidence is mounting that numerous miRNAs are involved in regulating drug resistance, especially in colorectal cancer 9,10. In our study, a miRNA microarray array analysis was conducted to identify the aberrant miRNAs that can regulate the tolerance of CRC cells to oxaliplatin. We discovered a single miRNA, miR-27b-3p, which was greatly downregulated in both two oxaliplatin-resistant cell lines. Due to the different cellular contexts of tumors, miR-27b-3p has been reported to serve as an oncogene 11 or a tumor suppressor 12,13 in tumor progression. Interestingly, previous studies suggested that miR-27b-3p could improve the anticancer effects of chemotherapeutic drugs in multiple human cancers 14. However, the mechanism of miR-27b-3p in regulating oxaliplatin resistance in CRC cells remains elusive.

Mounting evidence has demonstrated that anti-cancer therapies, including the cytotoxic chemotherapy, can induce cyto-protective autophagy in most cancer cells 15. Briefly, autophagy is a highly conserved cellular process during evolution, which is induced by diverse pathologies and cellular stresses containing nutrient deprivation, endoplasmic reticulum stress and hypoxia 16. Autophagy has also been involved in cancer resistance to multiple chemotherapeutic drugs, including cisplatin 17, doxorubicin 18, 5-Fu 19, and so on. Autophagy plays a vital role in regulating colorectal cancer chemoresistance, blocking of which will be developed as a promising therapy strategy for colorectal cancer treatment 10. By modulating key autophagy-related proteins expression, miRNA has an effect on regulating autophagy 10,20. More generally, it has attracted substantial attention that the contribution of modulation of autophagy is mediated by miRNAs in cancer therapy and drug resistance.

Here, we demonstrate the effects of miR-27b-3p on inhibiting autophagy and resensitizing chemoresistant cells to oxaliplatin. Moreover, we provided evidence showing that miR-27b-3p could target a key autophagy-related protein: ATG10, which is associated with tumorigenesis. A previous work has showed that miR-27b-3p is downregulated by c-Myc 21, and the relationship was confirmed in present study. In simple terms, we have demonstrated that the c-Myc/miR-27b-3p/ATG10 regulatory axis plays a vital role in regulating chemoresistance by activating the autophagy pathway in CRC.

## Materials and Methods

### MiRNA expression microarray

Total RNA extracted from SW480, HCT116, SW480-OxR and HCT116-OxR cells were used for Affymetrix miRNA microarray analysis (CapitalBio Corp, Beijing, China), and the process was described on the web site of CapitalBio (http://www.capitalbio.com).

### Tissue samples

Colorectal cancer tissues and adjacent normal tissues were also obtained from Tianjin Medical University Cancer Institute and Hospital (Tianjin, China). Written consent was provided by all the patients (or their guardians), and the Ethics Committee of Tianjin Medical University Cancer Institute and Hospital approved all aspects of this study. IHC and H&E staining were performed using paraffin-embedded sections of biopsies as described previously 22.

### Cell culture

Six human colorectal cancer cell lines HCT116, SW480, HT29, SW620, Caco2 and LOVO were obtained from the Shanghai Institute of Cell Biology (Shanghai, China).The related oxaliplatin-resistant cell lines SW480-OxR and HCT116-OxR were generated by continuous exposure to increasing concentrations of oxaliplatin for a 10-month period as described previously 23. We performed cytotoxicity testing to confirm that chemoresistance could be stable for about 4 weeks without oxaliplatin exposure. The oxaliplatin-resistant cell lines were used at no higher than 15 passages from creation. All cells were cultured in the appropriate medium (RPMI-1640 for HT29, SW620, SW480 and SW480-OxR cells; DMEM for Caco2, LOVO, HCT116 and HCT116-OxR cells) supplemented with 10% FBS (Gibco, Carlsbad, CA, USA) in a humidified atmosphere with 5% CO2 at 37 °C.

### Reagents and antibodies

Oxaliplatin (S1224) and CQ (S4330) were purchased from Selleck Chemicals (Houston, TX, USA). The antibodies used for western blot were as follows: anti-c-Myc antibody (sc-40, 1:1500, Santa Cruz, CA, USA), anti-ACTB antibody (sc-10731, 1:2000, Santa Cruz), anti-p62 antibody (66184-1-Ig, 1:1000, Proteintech, IL, USA), anti-LC3 antibody (14600-1-AP, 1:1000, Proteintech), anti-cleaved PARP antibody (#5625, 1:1000, Cell Signaling Technology, MA, USA), anti-cleaved-caspase 3 antibody (#9664, 1:1000, Cell Signaling Technology), anti-ATG10 antibody (DF8366, 1:1000, Affinity, OH, USA), anti-γ-H2AX (ab2893; Abcam, Cambridge, MA, USA), anti-ATG2A antibody (23226-1-AP, 1:1000, Proteintech), anti-ATG2B antibody (251551-1-AP, Proteintech), anti-ATG4C antibody (20382-1-AP, 1:1000, Proteintech).

### Transfection

MiRNA mimics, inhibitors, negative controls (NC or NC inhibitors), lentiviruses to overexpress or knowndown miR-27b-3p were purchased from GenePharma (Shanghai, China). To overexpress or knock down the expression level of proteins, gene-specific overexpression plasmids (FulenGen, Guangzhou, China) or siRNAs (GenePharma) were transfected into cells. The siRNA sequences are listed in Supplementary Table S1.

### RNA isolation and real-time quantitative PCR (qRT-PCR)

TRIzol reagent (Sigma, St. Louis, USA) was used for extracting total RNA from CRC tissues and cells. Total RNA was extracted from paraffin-embedded of cancer tissues using an RNA pre Pure FFPE Kit (Tiangen, Beijing, China) following the manufacturer's protocol. qRT-PCR for miRNAs and mRNAs were performed as described previously 22. U6 snRNA or ACTB was used as the internal control for miRNAs or protein-coding genes, respectively. The sequences of the primers are listed in Supplementary Table S1.

### Luciferase reporter assay

Luciferase vectors were purchased from Genescript (Nanjing, China). Briefly, for miRNA binding site assays, luciferase reporter gene plasmids harboring the wild-type 3'UTR of ATG10, ATG4C, ATG2A or ATG2B were constructed. We also constructed a mutant 3'UTR of ATG10, which was mutated from ACUGUGA to TGACACT. For the miR-27B promoter activity assay, miR-27B promoter regions containing different c-Myc binding sites were inserted into pGL3-Basic reporter gene vectors from Genescript (Nanjing, China). We cotransfected SW480-OxR cells with luciferase vectors, small RNA oligos and a β-galactosidase expression plasmid (Ambion, Carlsbad, CA, USA). Twenty-four hours after transfection, Luciferase activity was measured using a luciferase assay kit (Promega, USA).

### Xenograft studies

SW480, SW480-OxR, HCT116 and HCT116-OxR cells were infected with the miR-27b overexpression lentivirus, or negative control lentivirus, according to the manufacturer's instructions. In addition, SW480 and HCT116 were also transfected with miR-27b inhibitor sponge lentivirus or negative control lentivirus. The cells were then collected for quantitative RT-PCR, or animal experiments. To explore the role of miR-27b-3p in CRC chemoresistance in vivo, we designed twelve groups (n = 5): SW480-OxR /control, SW480-OxR /control + OXA, SW480-OxR /miR-27b + OXA, SW480/control, SW480 /control + OXA, SW480/miR-27b inhibitor sponge + OXA. HCT116-OxR /control, HCT116-OxR /control + OXA, HCT116-OxR /miR-27b + OXA, HCT116/control, HCT116 /control + OXA, HCT116/miR-27b inhibitor sponge + OXA. Equal numbers of cells (5 × 10^6^) were subcutaneously injected into each mouse to establish the CRC xenograft model. One week later, mice received an intraperitoneal injection of PBS or oxaliplatin (10 mg/kg) once per weekly for 3 weeks. On day 28, the animals were euthanized and tumors were removed. In the following experiments, SW480/control, SW480/miR-27b, HCT116/control, HCT116/miR-27b were subcutaneously injected into each mouse to establish the CRC xenograft, and 5-Fu (50 mg/kg) was used to treat the mice. Xenograft tumor tissues were removed for haematoxylin and eosin (H&E) staining or immunohistochemical (IHC) staining for Ki-67, ATG10 and cleaved-caspase 3.All the procedures were performed on the basis of the guidelines of the Laboratory Animal Ethics Committee of Tianjin Medical University Cancer Institute and Hospital.

### Assessment of cell proliferation assay and apoptosis

The cell proliferation assay was performed as described before 24. Briefly, CRC cells were transfected as indicated. After 12 h, 1 × 10^4^ cells were seeded into 96-well plates, and medium containing oxaliplatin was added to each well. After 48h incubation, a CCK8 (Dojindo, Japan) assay was performed. The IC_50_ and the cell viability rate were calculated. Apoptosis analysis was performed using an Annexin V FITC/PI double staining assay (BD Biosciences, San Jose, CA) following the manufacturer's protocol.

### Transmission electron microscopy (TEM)

CRC cells were treated as indicated and harvested in a 1.5 ml microcentrifuge tube. For electron microscopy, cells were fixed with 2.5% glutaraldehyde diluted in phosphate buffer and stored at 4 °C until embedding, followed by staining with 1% OsO4. After dehydration in an increasing gradient alcohol series, thin sections were stained with 3% lead citrate-uranyl acetate and photographed with a JEM-1100 transmission electron microscope (JEOL, Tokyo, Japan).

### GFP-LC3 analysis

CRC cells were transfected with GFP-LC3 vectors (HanBio Technology, Shanghai, China) and cotreated as indicated. GFP-expressing spots, which were indicated by green puncta, were imaged by a Nikon confocal microscope (Nikon, Tokyo, Japan) equipped with a 100× oil immersion objective. The number of spots per cell was determined by dividing the total number of spots by the number of nuclei in each field.

### In situ hybridization and and immunofluorescence (IF)

In situ hybridization (ISH) for miR-27b-3p was performed on fixed paraffin-embedded sections of biopsies from CRC samples by Roche Technology via standard protocols. The oligonucleotide probes complementary to miR-27b-3p were purchased from the GenePharma. Immunofluorescence was done as before 22. Briefly, Cells were treated as indicated, then were fixed and incubated with primary, secondary antibodies, respectively, and DAPI for nuclear staining. Images were recorded using microscope.

### Chromatin immunoprecipitation (ChIP) assay

The ChIP assay was performed with a commercial kit (Beyotime, Shanghai, China), following the manufacturer's instructions. Briefly, after fragmentation of genomic DNA extracted from SW480-OxR cells, an anti-c-Myc antibody (Santa Cruz; sc-40) was used to immunoprecipitate c-Myc-chromatin complexes, and anti-IgG (Santa Cruz) was used as the negative control antibody. PCR was performed to amplify the ChIP products, and the amplification products were then separated on 2.5% agarose gels. The primers used for amplification are listed in Supplementary Table S1.

### Statistical analysis

The data are presented as the means ±S.E.M. of at least three independent experiments. GraphPad Prism Software (GraphPad) and Statistical Program for Social Sciences 20.0 software (SPSS) were used for statistical analyses. Differences between groups were analyzed using Student's t-test or x^2^ test. The Pearson correlation test were calculated to estimate the correlations. The Kaplan-Meier survival function was calculated and compared with a log-rank test. Analysis of univariate or multivariate Cox proportional hazards regression was conducted with the hazard ratios and p values indicated. Statistically significance was defined: *p<0.05, **p<0.01 and ***p<0.001.

## Results

### Oxaliplatin-resistant colorectal cancer cells express decreased levels of miR-27b-3p

**Figure 1:**
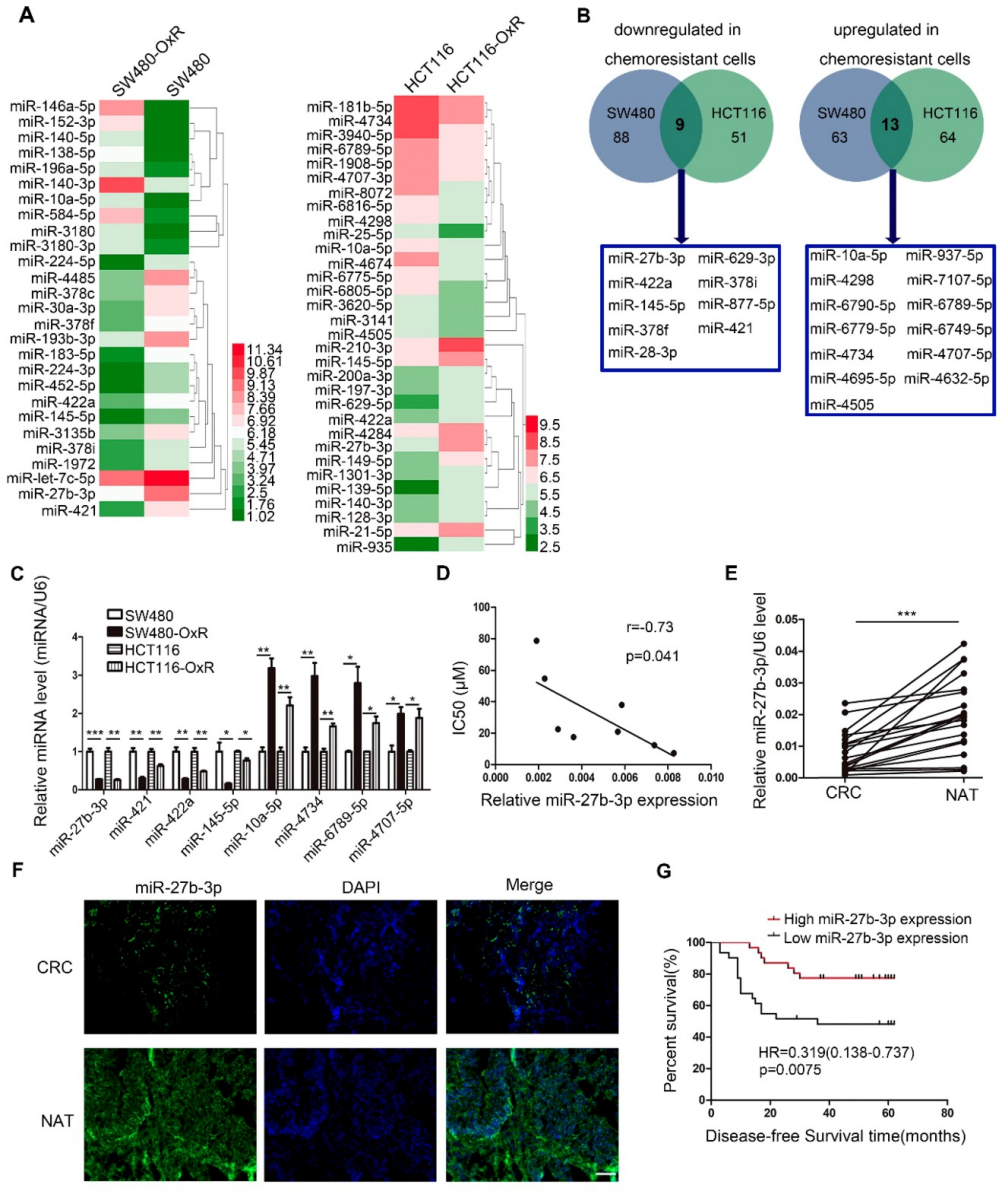
Oxaliplatin-resistant colorectal cancer cells express decreased levels of miR-27b-3p. (A) Different miRNA expressions levels in parental cells (SW480 and HCT116) and chemoresistant cells (SW480-OxR and HCT116-OxR) were determined by using the miRNA microarray. (B) Twenty-two miRNAs were dysregulated in oxaliplatin-resistant cells relative to their expression in the corresponding parental cells. (C) The relative levels of miRNAs in SW480, HCT116, SW480-OxR and HCT116-OxR cell lines were determined using qRT-PCR. (D) The correlation between the expression level of miR-27b-3p and IC_50_ for oxaliplatin in 8 CRC cell lines (SW480, HCT116, SW480-OxR, HCT116-OxR, SW620, Caco2, HT-29 and LOVO) was shown. (E) MiR-27b-3p expression levels were decreased in human colorectal cancer samples compared with those in the paired noncancerous tissues (n=20). (F) Representative images of the expression of miR-27b-3p in paired tissues using ISH. Scale bars: 100 μm. (G) Kaplan-Meier plots for investigating the correlation of miR-27b-3p expression level with disease-free survival (DFS). Patients were split into the high- and low-expression groups by the mean expression level of the miR-27b-3p (n=62; log-rank test). *p < 0.05, **p < 0.01, ***p < 0.001.

To screen miRNAs that can participate in the response of colorectal cancer cells to oxaliplatin, we generated two drug resistant cell lines (SW480-OxR and HCT116-OxR) (Supplementary Figure S1A and B). Next, miRNA microarray technology showed that the expression levels of various miRNAs were changed between oxaliplatin-resistant and the parental cells (Figure 1A and Supplementary Table S2). Figure 1B lists all miRNAs with common aberrant expression in oxaliplatin-resistant cells compared to that in the corresponding parental cells. Furthermore, the qRT-PCR analysis results confirmed that the expression levels of eight miRNAs were different (Figure 1C). Then, we transfected the mimics or inhibitors of preselected miRNAs into oxaliplatin-resistant cells to detect the function of these miRNAs in regulating chemoresistance. Among all miRNAs identified to regulate drug resistance, the effect of miR-27b-3p on reversing chemoresistance was the most powerful (Supplementary Table S3). In addition, The Pearson correlation analysis showed a significant negative correlation between the miR-27b-3p level and drug resistance in eight CRC cell lines (Figure 1D).

Moreover, we found that the expression of miR-27b-3p was dramatically decreased in cancer tissues compared to noncancerous tissues (Figure 1E and F and Supplementary Table S4). In addition, we detected the expression of miR-27b-3p in 62 colorectal cancer patients who received oxaliplatin-based chemotherapy. The results showed that miR-27b-3p expression was significantly downregulated in patients with recurrence compared to that in patients without recurrence (Supplementary Figure S1C). Kaplan-Meier survival analysis suggested that low levels of miR-27b-3p was significantly associated with shorter disease-free survival (DFS) time (Figure 1G and Supplementary Table S5). Furthermore, univariate and multivariate Cox regression analyses revealed that low levels of miR-27b-3p was an independent prognostic factor for poor prognosis of patients with colorectal cancer (Supplementary Figure S1D and E and Table S6).Our data indicate that miR-27b-3p is clinically associated with colorectal cancer recurrence and patient outcome.

### MiR-27b-3p reverses the chemoresistance of colorectal cancer cells

**Figure 2:**
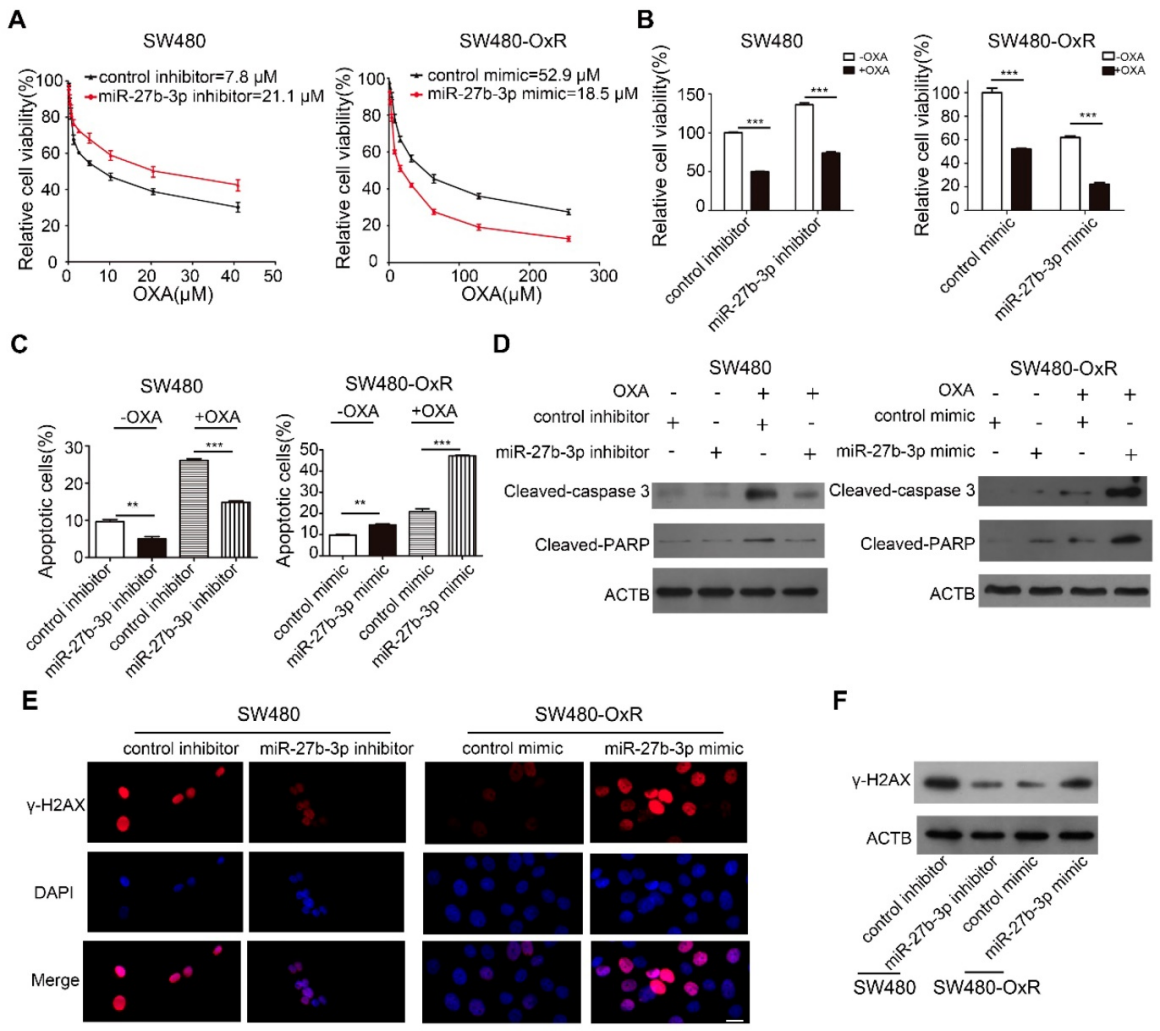
MiR-27b-3p reverses the chemoresistance of colorectal cancer cells. (A) Growth curves of SW480 cells (left) and SW480-OxR cells (right) after transfection as indicated. (B) The CCK8 assay showed a change in cell viability in response to oxaliplatin after transfection of SW480 cells (left) and SW480-OxR cells (right). (C) Cell apoptotic rates of SW480 (left) and SW480-OxR (right) cells were detected by flow cytometry. (D) Cleaved-caspase 3 and PARP expression were observed by western blot in SW480 cells (left) and SW480-OxR cells(right).(E) Formation of γ-H2AX foci was observed in SW480 (left) and SW480-OxR (right) cells. Scale bars: 20 μm. (F) γ-H2AX expression was detected by western blot in SW480 and SW480-OxR cells. **p < 0.01, ***p < 0.001.

Based on the accumulated data, we sought to examine the effect of miR-27b-3p on CRC cells chemoresistance in vitro. We inhibited miR-27b-3p expression in oxaliplatin-sensitive cells and overexpressed miR-27b-3p in oxaliplatin-resistant cells, respectively (Supplementary Figure S2A and B). Subsequently, the growth curves showed that miR-27b-3p inhibitor increased the IC_50_ of oxaliplatin in oxaliplatin-sensitive cells. On contrary, the IC_50_ of oxaliplatin concomitantly decreased in miR-27b-3p-overexpressing cells (Figure 2A and Supplementary Figure S2C). Next, by adding oxaliplatin to the corresponding cells, we found that overexpression of miR-27b-3p could enhance the effect of oxaliplatin on inhibiting cell proliferation, while the miR-27b-3p inhibitor had contrasting effects (Figure 2B and Supplementary Figure S2D). Apoptosis is believed to be an important indicator of the antitumor effects of platinum-based chemotherapy 24. To further investigate the role of miR-27b-3p in oxaliplatin-induced apoptosis, we measured the apoptosis rate in colorectal cancer cells. The results showed that the inhibition of miR-27b-3p could reduce apoptosis of oxaliplatin-sensitive cells, whereas overexpression of miR-27b-3p accelerated the apoptosis of oxaliplatin-resistant cells. (Figure 2C and Supplementary Figure S2E and F). Additionally, western blot confirmed that oxaliplatin increased the expression levels of cleaved-caspase 3 and PARP, and these effects were enhanced by miR-27b-3p overexpression, while inhibition of miR-27b-3p blocked the effects (Figure 2D and Supplementary Figure S2G). Oxaliplatin could cause DNA DSBs, are associated with the formation of γ-H2AX 14. Indeed, overexpression of miR-27b-3p resulted in the accumulation of γ-H2AX in oxaliplatin-resistant cells. Conversely, miR-27b-3p down-regulation reduced the level of oxaliplatin-induced foci formation of γ-H2AX in oxaliplatin-sensitive cells (Figure 2E and F and Supplementary Figure S2H and I).

In addition to oxaliplatin, 5-Fu also serves as the backbone of systemic combination chemotherapy in CRC treatment 25. Thus, we investigated whether miR-27b-3p could affect proliferation and apoptosis of CRC cells, when exposed to 5-Fu. Following treatment with 5-Fu, miR-27b-3p could inhibit proliferation (Supplementary Figure S3A and B) and enhance apoptosis (Supplementary Figure S3C-E). What's more, miR-27b-3p could markedly increase the sensitivity of colorectal cancer cells to 5-Fu in vivo (Supplementary Figure S3F-I). Thus, miR-27b-3p may enhance the sensitivity of CRC cells to chemotherapeutic agents.

### MiR-27b-3p suppresses tumor growth when combined with oxaliplatin in vivo

**Figure 3:**
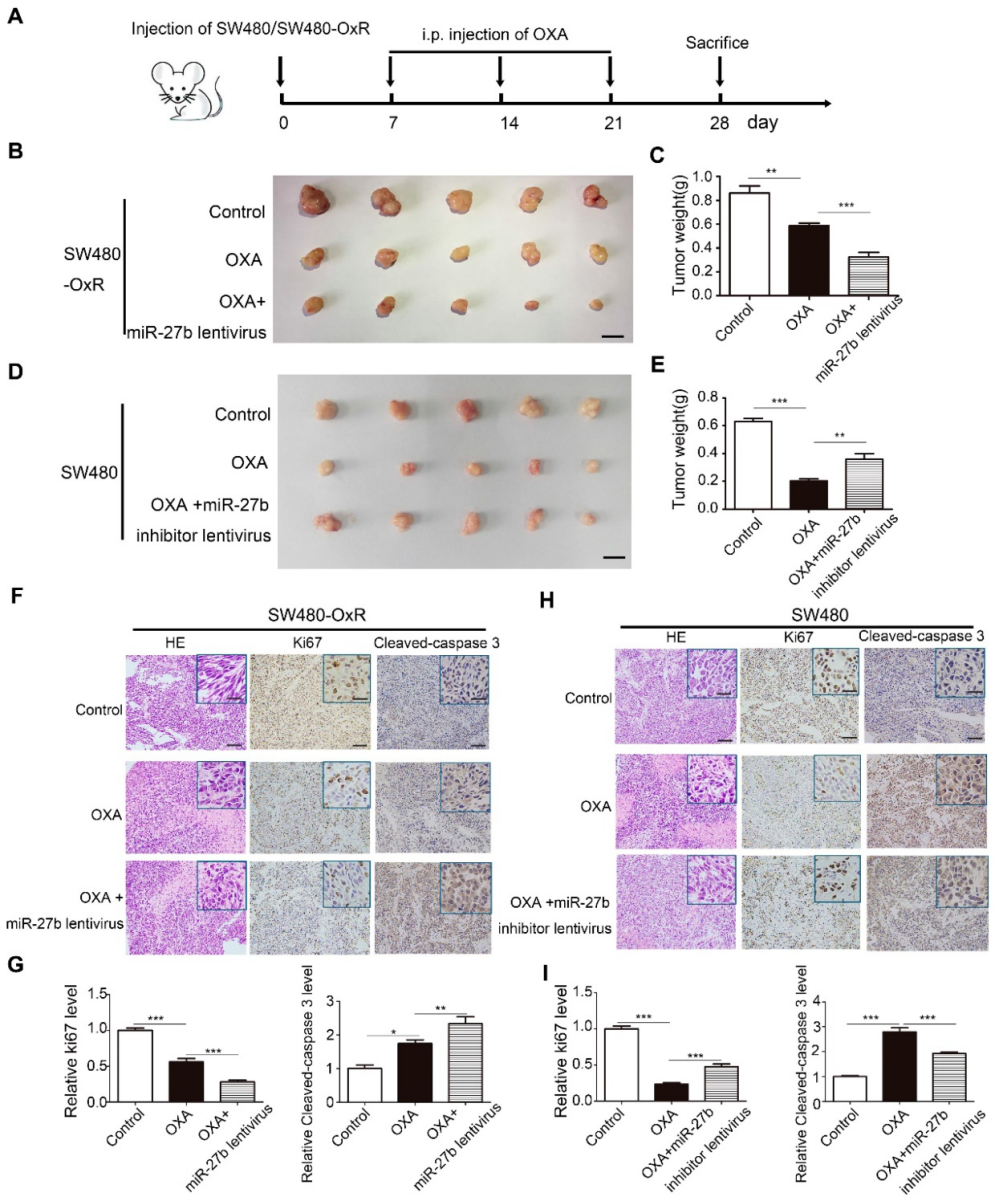
MiR-27b-3p suppresses tumor growth combined with oxaliplatin in vivo. (A) A schematic outline of the experimental design. (B) Representative images of tumors in nude mice bearing SW480-OxR cells in different groups (n= 5 for each group). Scale bars: 1 cm. (C) Tumor weights were measured in different groups. (D) Representative images of tumors in nude mice bearing SW480 cells in different groups (n= 5 for each group). Scale bars: 1 cm. (E) Tumor weights were measured in different groups, (F) Representative images of tumor samples derived from SW480-OxR group that were stained with H&E (left) and immunohistochemistry of Ki67 (middle) and cleaved-caspase 3 (right). Scale bars: 100 μm; (insets) 25 μm. (G) Statistical analysis of Ki-67 and cleaved-caspase 3 protein levels in (F). (H) Representative images of tumor samples derived from SW480 group that were stained with H&E (left) and immunohistochemistry of Ki67 (middle) and cleaved-caspase 3 (right). Scale bars: 100 μm; (insets) 25 μm. (I) Statistical analysis of Ki-67 and cleaved-caspase 3 protein levels in (H). *p < 0.05, **p < 0.01, ***p < 0.001.

To assess the effect of miR-27b-3p combined with oxaliplatin on tumor growth in vivo, we stably transfected SW480-OxR and HCT116-OxR cells with lentivirus overexpressing miR-27b-3p or with lentivirus expressing miR-NC (Supplementary Figure S4A). In addition, we also stably transfected SW480 and HCT116 cells with lentivirus expressing a miR-27b-3p inhibitor sponge or miR-NC (Supplementary Figure S4A). Because miR-27b lentivirus could overexpress both miR-27b-3p and miR-27b-5p, we further examined the expression level of miR-27b-5p in CRC cell lines. The expression of miR-27b-5p was much lower than that of miR-27b-3p in miRNA microarray, and qRT-PCR further conformed the result (Supplementary Figure S4B and Table S2). We then overexpressed miR-27b-5p in SW480-OxR and HCT116-OxR cells, and miR-27b-5p could not significantly enhance the effect of oxaliplatin on inhibiting cell proliferation (Supplementary Figure S4C and D). In the CRC xenograft mouse models, SW480-OxR cells and SW480 cells were subcutaneously transplanted into nude mice, and then offer treatment with oxaliplatin, as shown in Figure 3A. The results showed that SW480-OxR and HCT116-OxR cells stably overexpressing miR-27b-3p were more sensitive to oxaliplatin therapy than control group (Figure 3B and C and Supplementary Figure S5A and B). Oppositely, inhibition of miR-27b-3p in SW480 and HCT116 cells weakened the effect of oxaliplatin on inhibiting tumor growth (Figure 3D and E and Supplementary Figure S5C and D). qRT-PCR showed that oxaliplatin treatment decreased miR-27b-3p level, which were recovered by expression of miR-27b-3p in SW480-OxR and HCT116-OxR xenograft tumors. In contrast, miR-27b-3p expression was downregulated by miR-27b-3p sponge inhibitor in SW480 and HCT116 xenograft tumors (Supplementary Figure S5E). Additionally, the reduction of Ki67 and upregulation of cleaved-caspase 3 were detected in SW480-OxR and HCT116-OxR cells treated with the combination of miR-27b-3p-overexpressing lentivirus and oxaliplatin (Figure 3F and G and Supplementary Figure S5F and G), while SW480 and HCT116 cells stably expressing the miR-27b-3p inhibitor exhibited an increased Ki67 and reduced cleaved-caspase 3 level (Figure 3H and I and Supplementary Figure S5H and I). Altogether, these results strongly indicate that miR-27b-3p suppresses tumor growth and inhibits therapeutic resistance in vivo.

### MiR-27b-3p inhibits oxaliplatin-induced autophagy in chemoresistant CRC cells

**Figure 4:**
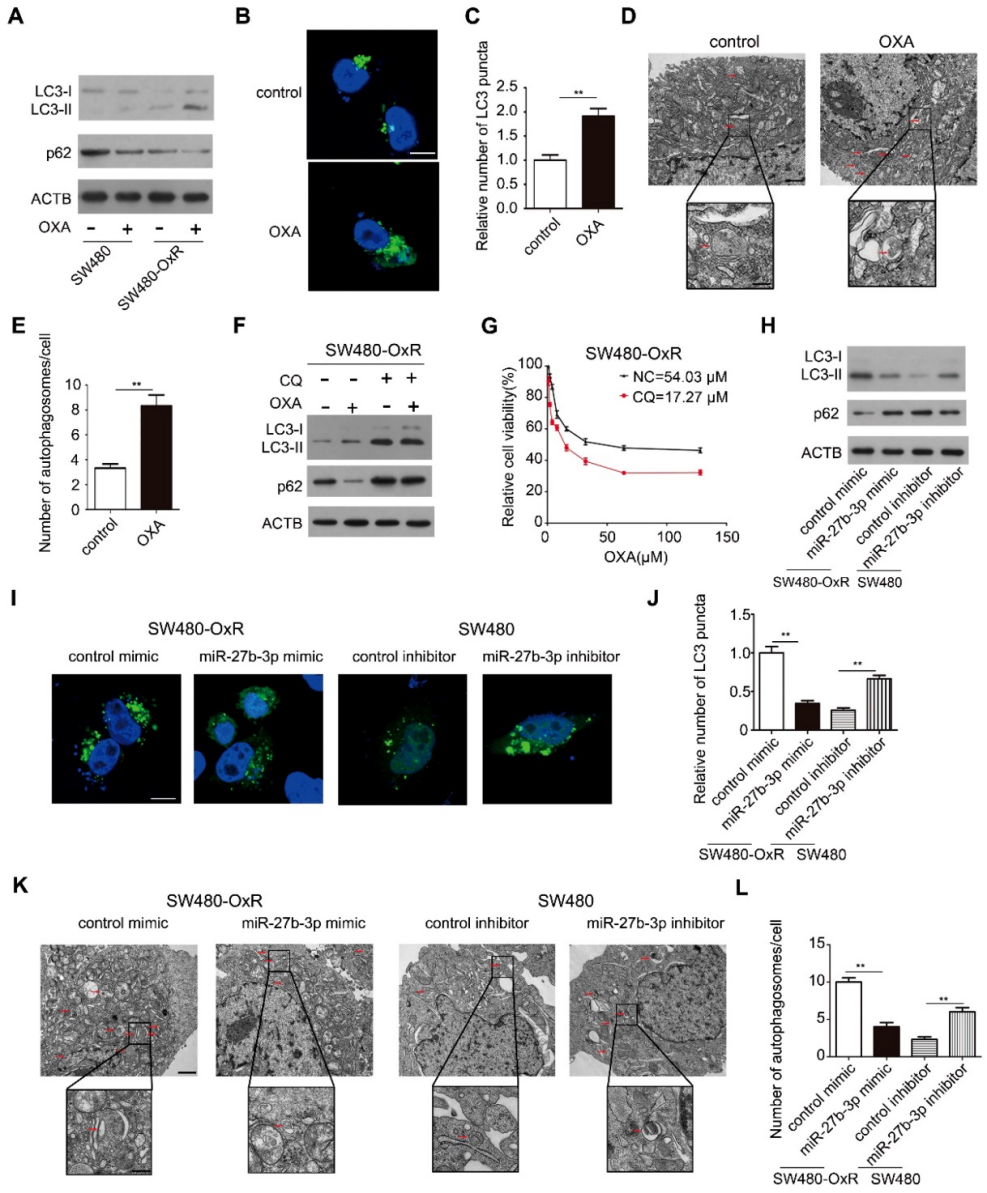
MiR-27b-3p inhibits autophagic activity in chemoresistant CRC cells. (A) Autophagy element expression levels were detected by western blot in SW480 and SW480-OxR cells cultured with oxaliplatin. (B and C) Confocal microscopic analysis was performed to observe green fluorescent LC3 puncta in SW480-OxR cells cultured with oxaliplatin. Representative images are shown in (B), and LC3 puncta per cell were quantified in (C). Scale bar: 10 μm. Autophagosomes were observed by transmission electron microscopy (TEM) in SW480-OxR cells cultured with oxaliplatin. Representative images are shown in (D), and autophagosomes per cell were quantified in (E). Scale bar: 1 μm; (insets) 250 nm. (F)Western blot was performed in SW480-OxR cells treated with oxaliplatin in the presence of CQ. (G) IC_50_ for oxaliplatin in SW480-OxR cells in the presence or absence of CQ. (H-L) SW480-OxR and SW480 cells were transfected with mimics or inhibitor of miR-27b-3p, respectively. After culturing with oxaliplatin, (H) autophagy element expression levels were detected by western blot, (I) green fluorescent LC3 puncta were observed under confocal microscope, (K) autophagosomes were observed by TEM, respectively. LC3 puncta per cell were quantified in (J). Scale bar: 10 μm. Autophagosomes per cell were quantified in (L). Scale bar: 1 μm; (insets) 250 nm. **p < 0.01.

Considering that autophagy can protect cancer cells from cytotoxic drugs, we explored whether autophagy participated in chemoresistance to oxaliplatin in colorectal cancer. Thus, we measured the LC3 and p62 levels, which are the widely used markers of autophagy 15. Remarkably, oxaliplatin-resistant cells showed higher LC3-II protein levels and lower p62 protein levels than the corresponding parental cells after treatment with oxaliplatin, suggesting that autophagic flux was induced when chemoresistance occurred (Figure 4A and Supplementary Figure S6A).Consistent with this result, oxaliplatin treatment significantly induced the formation of LC3 puncta (Figure 4B and C) and autophagosomes (Figure 4D and E) in SW480-OxR cells. Next, we sought to explore whether chemotherapy-induced autophagy has an effect on the efficacy of chemotherapy. Thus, we co-treated SW480-OxR cells with chloroquine (CQ) and oxaliplatin, and found that CQ enhanced the antitumor activity of oxaliplatin, as evidenced by the decreased IC_50_ (Figure 4F and G).

We next investigated the role of miR-27b-3p in autophagic activity. Overexpression of miR-27b-3p resulted in reducing level of LC3-II and enhancing level of p62 in oxaliplatin-resistant cells (Figure 4H and Supplementary Figure S6B). In contrast, in oxaliplatin-sensitive cells with miR-27b-3p suppression, the level of LC3-II was increased, while the level of p62 was decreased (Figure 4H and Supplementary Figure S6B). Overexpression of miR-27b-3p has diminished the numbers of LC3 puncta, whereas suppression of miR-27b-3p led to an increase in the number of LC3 puncta (Figure 4I and J and Supplementary Figure S6C and D). In addition, upregulation of miR-27b-3p blocked the formation of autophagosomes and that inhibition of miR-27b-3p enhanced the formation of autophagosomes (Figure 4K and L). Collectively, our data indicate that chemoresistant cells show enhanced autophagy activity when compared to the corresponding parental cells, and that miR-27b-3p inhibits autophagic activity.

### Identification of ATG10 as a direct target of miR-27b-3p

**Figure 5:**
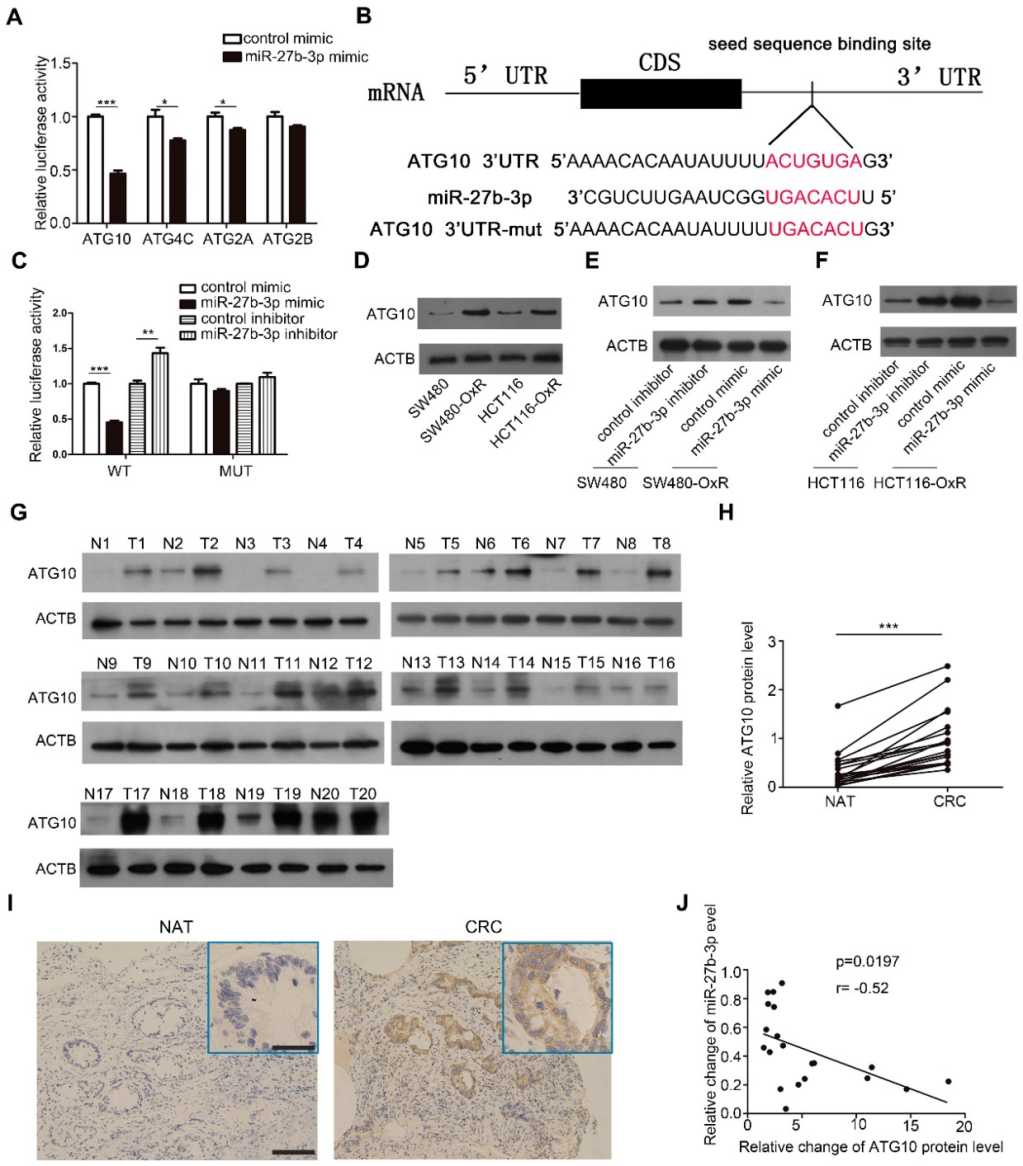
Identification of ATG10 as a direct target of miR-27b-3p. (A)Luciferase assays showing suppression of luciferase activity of candidate genes by miR-27b-3p in SW480-OxR cells. (B) Schematic of the hypothetical duplexes formed by miR-27b-3p and the 3'-UTR of ATG10. (C) Relative luciferase activity in SW480-OxR cells transfected with the miR-27b-3p mimic or inhibitor. (D) Western blot showing the expression levels of ATG10 in four CRC cell lines. (E and F) Western blot analysis was performed to measure the expression level of ATG10 in oxaliplatin-resistant cells transfected with the miR-27b-3p mimic and the corresponding parental cells transfected with the miR-27b-3p inhibitor. (G) Protein levels of ATG10 were measured in 20 pairs of samples using western blot as previously described. (H) The levels of ATG10 protein expression were measured. (I) Representative images of tumor samples that were stained for ATG10 by IHC. Scale bar: 100 μm; (insets) 25 μm. (J) The correction between the fold changes in the expression of miR-27b-3p and the ATG10 protein in human CRC tissue pairs (n=20). *p<0.05, **p<0.01, ***p<0.001.

To investigate the target gene involved in mediating the effect of miR-27b-3p on modulating autophagy, we using a combination of three prediction softwares: TargetScan 26, miRanda 27 and miRPathDB 28, and we selected four candidate genes, namely, ATG10, ATG4C, ATG2A and ATG2B. Among these 4 genes, ATG10 exhibited the most significant reduction in luciferase activity when ectopic miR-27b-3p was expressed in SW480-OxR cells (Figure 5A). By blocking the conversion of LC3-I to LC3-II, ATG10 plays a vital role in regulating autophagy 29. However, the function of ATG10 in regulating drug responses is unclear. The predicted interactions between miR-27b-3p and the 3ʹ-UTR of ATG10 are shown in Figure 5B. To further verify that ATG10 is a direct target of miR-27b-3p, we constructed a luciferase reporter vector containing wild-type or mutant 3' UTR fragments harboring the miR-27b-3p binding site of ATG10. The results showed that ectopic expression of miR-27b-3p significantly reduced the fluorescence intensity, whereas downregulation of miR-27b-3p enhanced the luciferase activity. When the binding site of miR-27b-3p was mutated, miR-27b-3p had no influence on the luciferase activity (Figure 5C).

Furthermore, western blot showed that ATG10 was the most significantly upregulated protein in oxaliplatin-resistant colorectal cancer cells (Figure 5D and Supplementary Figure S7A). Overexpression of miR-27b-3p inhibited the expression of ATG10, whereas inhibition of miR-27b-3p increased the ATG10 protein level, respectively (Figure 5E and F). However, the mRNA levels of ATG10 in CRC cells have not been changed (Supplementary Figure S7B and C), which indicated that miR-27b-3p could regulate ATG10 expression at the post-transcriptional level. Furthermore, we measured the ATG10 levels in the abovementioned 20 pairs of CRC tissues and found that the ATG10 levels were significantly upregulated in CRC tissues than in the paired normal colorectal tissues (Figure 5G-I). The Pearson correlation analysis revealed that the expression level of ATG10 was significantly negatively related to the level of miR-27b-3p (Figure 5J). In addition, the expression of ATG10 was decreased after overexpression of miR-27b-3p (Supplementary Figure S7D and F) and increased after inhibition of miR-27b-3p (Supplementary Figure S7E and G) in the subcutaneous colorectal tumors. In sum, these results demonstrate that miR-27b-3p posttranscriptionally regulates the expression of ATG10.

### MiR-27b-3p enhances the sensitivity of CRC cells to oxaliplatin by inhibiting ATG10 and thereby inhibiting autophagy

**Figure 6:**
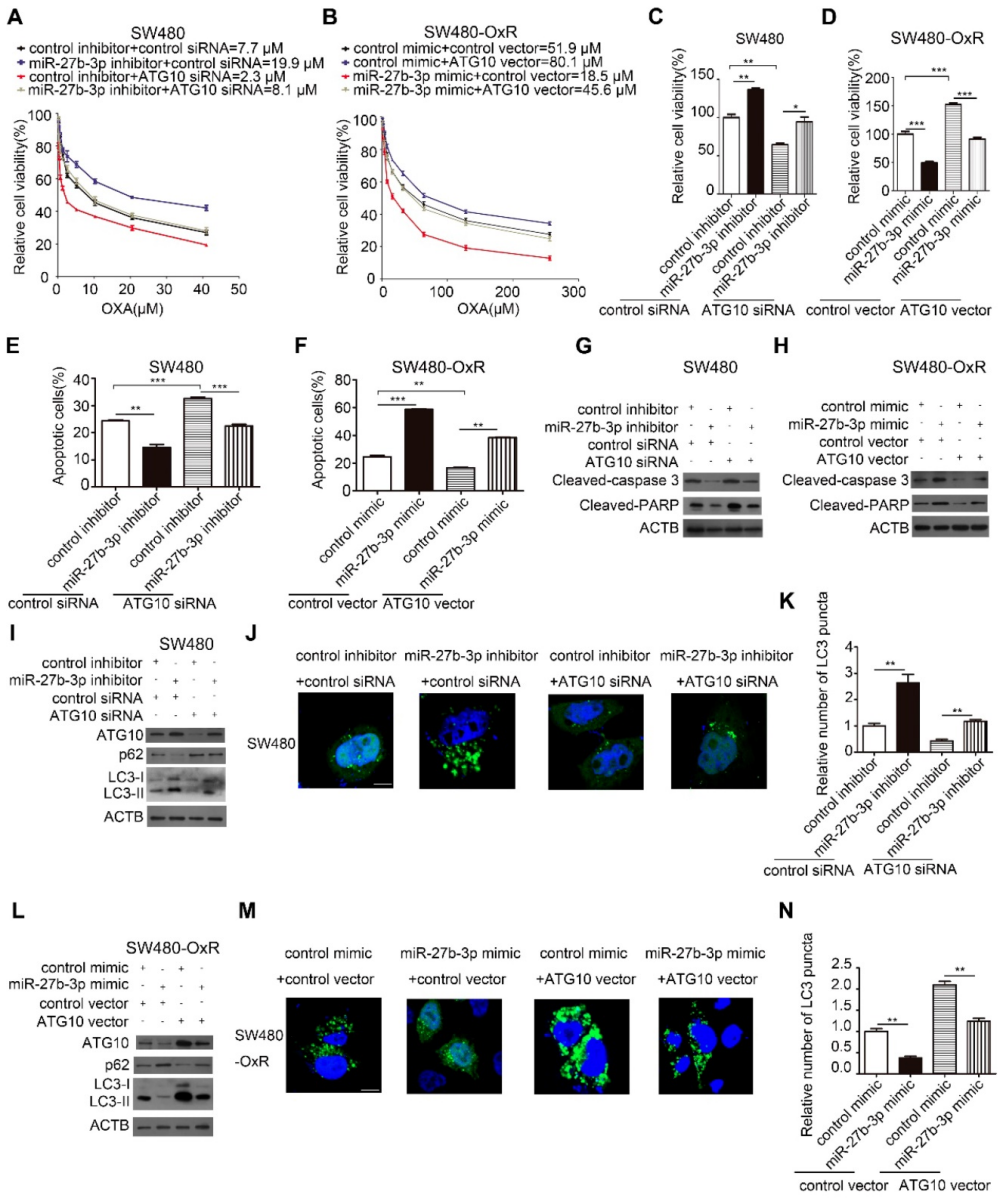
MiR-27b-3p enhances the sensitivity of CRC cells to oxaliplatin by inhibiting ATG10 and thereby inhibiting autophagy. (A and B) Growth curves of SW480 cells (A) and SW480-OxR cells (B) after transfection as indicated. (C and D) The CCK8 assay showed a change in cell viability in response to oxaliplatin after transfection of SW480 cells (C) and SW480-OxR cells (D). (E and F) Apoptosis was detected by flow cytometry in SW480 cells (E) and SW480-OxR cells (F) with the indicated modifications and then were incubated with oxaliplatin, respectively.(G and H) The protein levels of cleaved-caspase 3 and PARP in SW480 cells (G) and SW480-OxR cells (H) after transfection and then were stimulated with oxaliplatin. (I)Western blot analysis for autophagy element expression levels in SW480 cells, after treated as in (G). (J and K) Representative photographs of LC3 puncta (green) in SW480 cells with the indicated modifications (J). Quantification of LC3 puncta in the indicated SW480 cells (K). Scale bar: 10 μm. (L) Western blot analysis for autophagy elements expression levels in SW480-OxR cells, after treated as in (H). (M and N) Representative photographs of LC3 puncta (green) in SW480-OxR cells with the indicated modifications (M). Quantification of LC3 puncta in the indicated SW480-OxR cells (N). Scale bar: 10 μm. *p<0.05, **p<0.01, ***p<0.001.

Given the effect of autophagy on regulating drug resistance as described before, we hypothesized that miR-27b-3p reverses chemoresistance by attenuating autophagic activity through inhibiting ATG10. To further evaluate whether ATG10 mediates the function of miR-27b-3p in the autophagic process and chemoresistance, we performed a series of rescue experiments. The results showed that inhibition of ATG10 can suppress the proliferation and attenuate the drug resistance of SW480 cells (Figure 6A and C). In contrast, overexpression of ATG10 promoted cell proliferation and drug resistance of SW480-OxR cells (Figure 6B and D). Suppression of ATG10 abolished the enhancement of cell proliferation and drug resistance in SW480 cells induced by the miR-27b-3p inhibitor (Figure 6A and C). Moreover, the miR-27b-3p-induced inhibition of SW480-OxR cell proliferation and chemoresistance was reduced by the ATG10 overexpression vector (Figure 6B and D). In combination with oxaliplatin, downregulation of ATG10 increased oxaliplatin-induced apoptosis (Figure 6E and Supplementary Figure S8) and the protein levels of cleaved-caspase 3 and PARP (Figure 6G) in SW480 cells. More importantly, the attenuation of SW480 cell apoptosis in response to oxaliplatin by the miR-27b-3p inhibitor was significantly reversed by ATG10 downregulation (Figure 6E and G and Supplementary Figure S8). In addition, we showed that ATG10 overexpression significantly reduced oxaliplatin-induced cell apoptosis (Figure 6F and Supplementary Figure S8) and the expression levels of cleaved-caspase 3 and PARP (Figure 6H) in SW480-OxR cells. Moreover, the increase in the oxaliplatin-induced cell apoptosis rate by the miR-27b-3p mimic was significantly reversed by ATG10 overexpression (Figure 6F and H and Supplementary Figure S8).

Next, we investigated the effect of ATG10 on autophagic activity in colorectal cancer cells. In SW480 cells, silencing of ATG10 attenuated the effect of the miR-27b-3p inhibitor on the protein levels of ATG10, p62 and LC3-II (Figure 6I) and the formation of LC3 puncta (Figure 6J and K). Conversely, in SW480-OxR cells, ATG10 overexpression diminished the inhibitory effect of miR-27b-3p on the protein levels of ATG10, p62 and LC3-II (Figure 6L) and the formation of LC3 puncta formation (Figure 6M and N). Collectively, these results suggest that miR-27b-3p inhibits CRC cell chemoresistance and autophagy by suppressing ATG10.

### Expression of miR-27b-3p is inhibited by c-Myc

**Figure 7:**
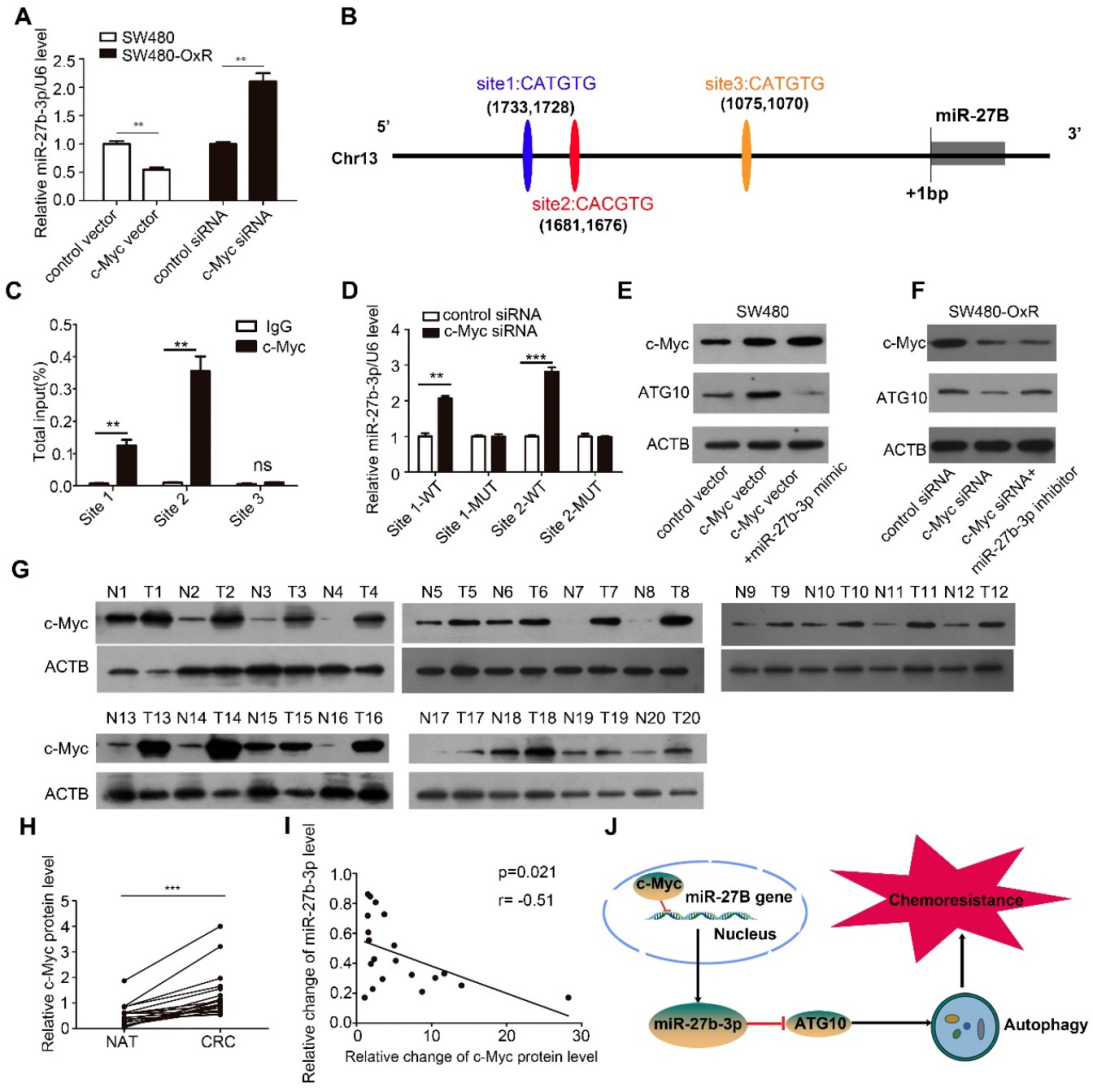
Expression of miR-27b-3p is inhibited by c-Myc. (A) The influence of c-Myc on the expression of miR-27b-3p. (B) Schematic showing the three putative c-Myc-binding motifs in the miR-27B promoter region. (C) ChIP assay for c-Myc occupancy on the miR-27B promoter region. (D) Luciferase reporter assays were performed to confirm the suppression of miR-27B promoter by c-Myc. (E and F) Western blot analysis of the c-Myc and ATG10 protein levels in SW480 cells (E) and SW480-OxR cells (F). (G and H) Western blot analysis of the c-Myc expression level in 20 pairs of CRC tissues and NATs. G: representative images; H: quantitative analysis (n=20). (I) The correction between the fold changes in the expression of miR-27b-3p and the c-Myc protein in CRC tissues as mentioned previously (n = 20). (J) Schematic of the c-Myc/miR-27b-3p/ATG10 axis in CRC. **p<0.01, ***p<0.001.

To investigate the mechanism underlying miR-27b-3p inhibition in chemoresistant CRC cells, we first measured the levels of pri-miR-27b in CRC cells and observed that they were also significantly lower in chemoresistant cells than in the corresponding parental cells, suggesting that miR-27b-3p is transcriptionally inhibited in chemoresistant cells (Supplementary Figure S9A). It has been widely reported that dysregulation of transcription factors (TFs), which can also regulate the expression of miRNA, is commonly involved in tumorigenesis 30. Interestingly, our previous work showed that c-Myc can transcriptionally downregulate miR-27b-3p 21. To examine the effect of c-Myc on regulating the miR-27b-3p, we overexpressed or knocked down c-Myc in SW480 cells or SW480-OxR cells, respectively (Supplementary Figure S9B). As shown in Figure 7A and Supplementary Figure S9C, the levels of mature miR-27b-3p and pri-miR-27b were markedly decreased or increased after transfection with the c-Myc overexpression plasmid or siRNA, respectively.

By binding to the E-box sequence CACGTG or CATGTG, c-Myc can downregulate the expression of miRNA at the transcriptional level 31. After analyzing the potential promoter region, we identified three putative c-Myc binding sites (Figure 7B). Chromatin immunoprecipitation technique showed that c-Myc was significantly recruited to the region around the binding sites 1 and 2 in SW480-OxR cells (Figure 7C and Supplementary Figure S9D). Subsequently, we cloned the binding sites 1 and 2 into the upstream region of a firefly luciferase reporter gene and then performed luciferase reporter assays in SW480-OxR cells. Silencing c-Myc enhanced luciferase activity in binding sites 1 and 2-containing plasmids, whereas luciferase activity was unaffected when the binding sites were mutated (Figure 7D).

Subsequently, we sought to investigate whether c-Myc could regulate ATG10 expression by inhibiting miR-27b-3p. As shown, transfection of the c-Myc vector increased the protein level of ATG10, which was attenuated by cotreatment with the miR-27b-3p mimic (Figure 7E). In contrast, inhibition of c-Myc expression significantly downregulated ATG10, and this effect was rescued following miR-27b-3p inhibitor transfection (Figure 7F). Meanwhile, we also found that transfection of the c-Myc vector decreased the level of miR-27b-3p, which was abolished by cotreatment with the miR-27b-3p mimic (Supplementary Figure S9E). In contrast, inhibition of c-Myc expression significantly upregulated miR-27b-3p, and this effect was attenuated by transfecting with miR-27b-3p inhibitor (Supplementary Figure S9F).Taken together, these results reveal that c-Myc indirectly upregulates the expression of ATG10 through inhibiting miR-27b-3p.

We then examined the c-Myc protein levels in the 20 abovementioned pairs of CRC tissues, and the results showed that the expression level of c-Myc was markedly elevated in the CRC tissues (Figure 7G and H). Notably, The Person correlation test showed a significant inverse relation between the levels of the c-Myc protein and miR-27b-3p in CRC tissues (Figure 7I). These results indicate that the decreased levels of the miR-27b-3p are, at least in part, attributed to the overexpression of c-Myc in CRC. Thus, we concluded that c-Myc specifically regulates miR-27b-3p expression transcriptionally and indirectly promotes ATG10 expression (Figure 7J).

## Discussion

Due to the target genes diversity and tissue type specificity of miRNAs, the specific regulatory functions of miRNAs have yet to be fully delineated 7. In our study, we have identified miR-27b-3p could significantly reduce cell chemoresistance and act as a promising marker for predicting prognosis in colorectal cancer patients receiving oxaliplatin-based chemotherapy. The role of miR-27b-3p in tumorigenesis remains to be elucidated. Previous reports showed that miR-27b-3p expression was elevated in certain human malignancies and that miR-27b-3p thus served as an oncogenic miRNA 11. However, it has also been reported that in colorectal cancer 32, gastric cancer 33, breast cancer 34, miR-27b-3p acts as a tumor suppressor. These conflicting conclusions from different studies may be due to the use of different cellular models. In terms of regulating drug-resistance, studies have shown that miR-27b-3p may increase drug resistance in anaplastic thyroid cancer 35. Conversely, in gastric cancer 36, breast cancer 34, and nasopharyngeal cancer 37, miR-27b-3p was reported to enhance the response to anticancer drugs such as doxorubicin and paclitaxel. However, the mechanism of miR-27b-3p regulating CRC chemoresistance requires further clarification.

Autophagy is known to play role in maintaining the survival of tumor cells under a variety of adverse conditions, including nutrient deficiency, chemotherapy and radiation treatment 15. Accumulating evidence suggests that regulating the autophagic activity could enhance the action of many antitumor agents, including oxaliplatin 10, cisplatin 17, doxorubicin 38 and 5-Fu 39. Thus, autophagy has been proposed as a potential drug target to reverse drug resistance. Previous reports have shown that a series of miRNAs regulate the drug resistance by modulating of autophagy 10. Notably, we reported that oxaliplatin-resistant cells showed increased autophagic activity compared to that in the corresponding parental cells. Interestingly, miR-27b-3p has been shown to inhibit PINK expression resulting in autophagy suppression 40. In the present study, we introduced miR-27b-3p as a novel autophagy regulator in CRC. In oxaliplatin-resistant CRC cells, overexpression of miR-27b-3p inhibited LC3-I to LC3-II conversion, GFP-LC3 accumulation and autophagosome synthesis in CRC cells. In summary, we introduced miR-27b-3p as a vital autophagy-regulating miRNA that acts as a tumor suppressor in CRC cells, by blocking autophagy to promote cell sensitivity to oxaliplatin.

ATG10 is an autophagic E2-like enzyme that interacts with ATG7 to recruit ATG12 and modulates the conversion of LC3-I to LC3-II 29. Thus, ATG10 plays a critical role in autophagosome formation. Emerging evidence has emphasized that ATG10 displayed higher expression level in tumors of malignancies such as CRC 41 and lung cancer 42. In addition, increased expression of ATG10 is positively linked with lymphovascular invasion and predicts decreased overall survival times 41. Further studies showed that ATG10 could promote tumor cells proliferation and malignant transformation 42. In our study, we demonstrated that miR-27b-3p regulates the expression of ATG10 at the posttranscriptionally level. Moreover, we assessed the effect of miR-27b-3p on inhibiting autophagy, cell proliferation, drug resistance, and even the growth of implanted tumors by suppressing ATG10. Therefore, our data may reveal the therapeutic potential of miR-27b-3p combined with chemotherapy.

In present study, we found decreased expression of miR-27b-3p in the oxaliplatin-resistant cells, but the mechanism requires further study. As a transcription factor, c-Myc is equipped with the ability to regulate tumor development in many types of human cancer by orchestrating gene expression 30. It has been shown that aberrant expression of c-Myc is a key driver of CRC progression 43. Numerous studies have revealed that c-Myc acts as either a transcriptional activator or inhibitor that modulates miRNA expression and contributes to cancer progression 30. Interestingly, our previous work showed that c-Myc could reduce the expression of miR-27b-3p, and we demonstrated this effect in CRC cells. MiR-27B is located within the 14th intron of its host gene, and far away from the host gene's transcriptional start sites (TSSs). It has been reported that intronic miRNAs located far away from their host TSSs may rely on independent novel TSSs to increase the speed and efficiency of transcription 44. Thus, the potential promoter region (approximately 2 kb upstream of the TSS) of miR-27B was analyzed, and the ChIP assay results suggested that c-Myc can bind to the promoter region of the miR-27B gene. Specifically, we found that in oxaliplatin-resistant colorectal cancer cells, the increased chemoresistance and autophagy are due to the enhanced expression of c-Myc, which upregulates the expression of ATG10 by suppressing miR-27b-3p. In fact, previous findings indicated that c-Myc can regulate tumor cell chemoresistance to antitumor platinum drugs 45. Furthermore, c-Myc can also trigger autophagy by inducing the expression of multiple ATG genes 46,47.

In addition to describing the biological importance of miR-27b-3p, the results of our study may be related to the clinical management of CRC patients. For CRC patients, capecitabine (and 5-Fu) has been widely used in combination with platinum-based chemotherapy, which can effectively inhibit tumors and initially improve the survival of patients 5. However, many patients eventually relapse due to the emergence of chemoresistance 4. Therefore, it is important to explore the regulatory mechanism of drug resistance and optimize current therapeutic strategies. Given that the expression level of miR-27b-3p is associated with the risk of CRC recurrence, detection of miR-27b-3p may be an effective approach to predict the response of patients to chemotherapy. Furthermore, our work suggested that combining miR-27b-3p with chemotherapeutic agents may elevate the therapeutic effect, providing a potential therapeutic avenue to control CRC, especially in patients who are resistant to chemotherapy.

## Conclusions

In summary, we found that c-Myc repressed the transcription of miR-27b-3p, thus indirectly regulated ATG10 expression. Furthermore, we observed that the c-Myc/miR-27b-3p/ATG10 regulatory axis could upregulate autophagy, leading to chemoresistance in colorectal cancer. Our findings providing a novel marker for sensory evaluation of chemotherapy and a potential therapeutic target to reverse chemoresistance in colorectal cancer.

## Supplementary Material

Supplementary figures and tables 1, 3-6.

Supplementary table 2.
