# Supplementary material for: The c-Myc/miR-27b-3p/ATG10 regulatory axis regulates chemoresistance in colorectal cancer
Source: Theranostics. 2020 Jan 12;10(5):1981–96. doi: 10.7150/thno.37621 (PMC7019154; doi:10.7150/thno.37621)
Supplement: Supplementary file 1 — Supplementary figures and tables 1, 3-6. [file thnov10p1981s1.pdf]

Supplementary Figures

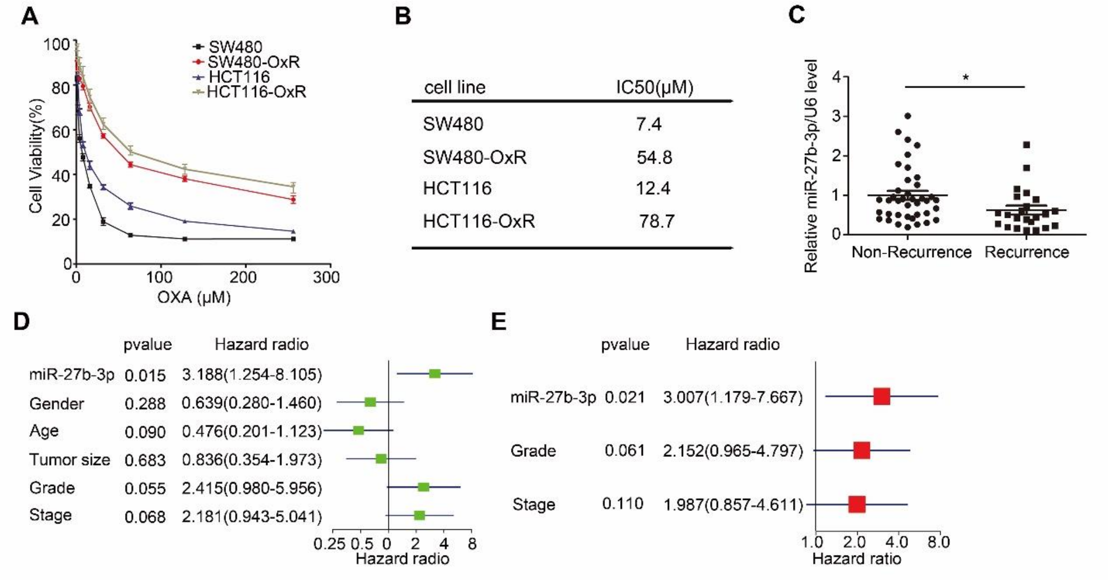

Figure S1. Establishment of oxaliplatin-resistant cells. (A) SW480-OxR and HCT116-OxR were significantly resistant to oxaliplatin in vitro, compared to their parental cells. Cells were treated with oxaliplatin for 48 h. (B) Comparison of drug resistance of oxaliplatin in different cell lines. (C) MiR-27b-3p expression levels were reduced in samples of patients with recurrence (n=23) compared with those in patients without recurrence (n=39). (D) Univariate analysis was performed in CRC patients received oxaliplatin-based chemotherapy. The bars correspond to 95% confidence intervals. (E) Multivariate analysis was performed in CRC patients received oxaliplatin-based chemotherapy. The bars correspond to 95% confidence intervals. *p < 0.05

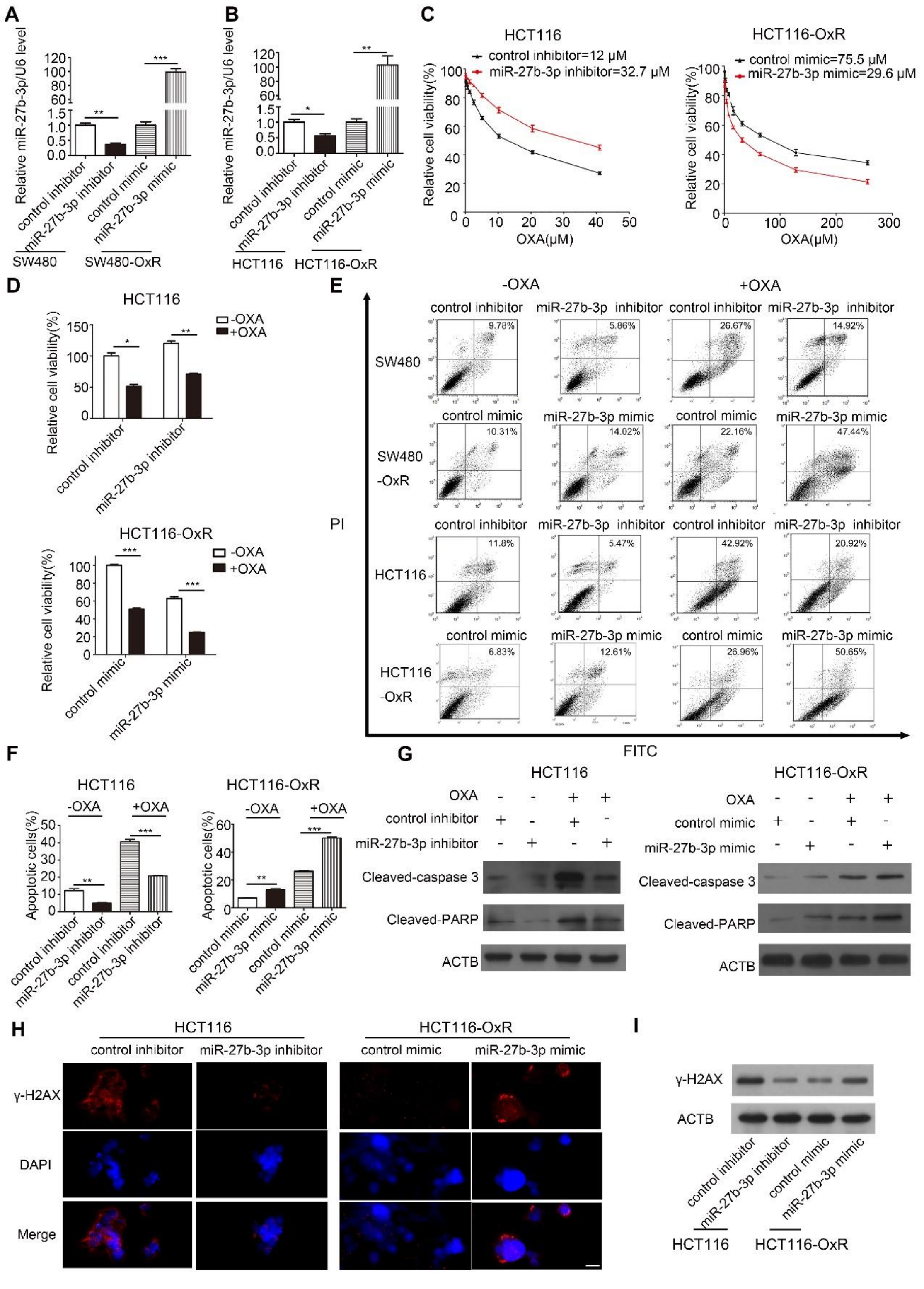

Figure S2. MiR-27b-3p reverses the chemoresistance of colorectal cancer cells. (A and B) qRT-PCR detected the expression levels of miR-27b-3p in CRC cells transfected as indicated. (C) After transfected with inhibitor (left) or mimic (right) for 24 h, CRC cells were treated with different concentrations of oxaliplatin (OXA) for 48 h. Then the cell viability was measured using CCK8 assay. (D) HCT116 and

HCT116-OxR cells were transfected as (C). Then the cells were treated with or without oxalipaltin for 48 h. Cell viability was measured by CCK8 assay. (E and F) Cell apoptotic rates of SW480, SW480-OxR, HCT116 and HCT116-OxR cells were detected by flow cytometry. (E): representative images; (F): quantitative analysis. (G) Cleaved-caspase 3 and PARP expression were observed by western blot in HCT116 cells (left) and HCT116-OxR cells (right). (H) Formation of γ-H2AX foci was observed in HCT116 (left) and HCT116-OxR (right) cells. Scale bars: 20 μm. (I) γ-H2AX expression was detected by western blot in HCT116 and HCT116-OxR cells. *$p < 0.05$, **$p < 0.01$, ***$p < 0.001$.

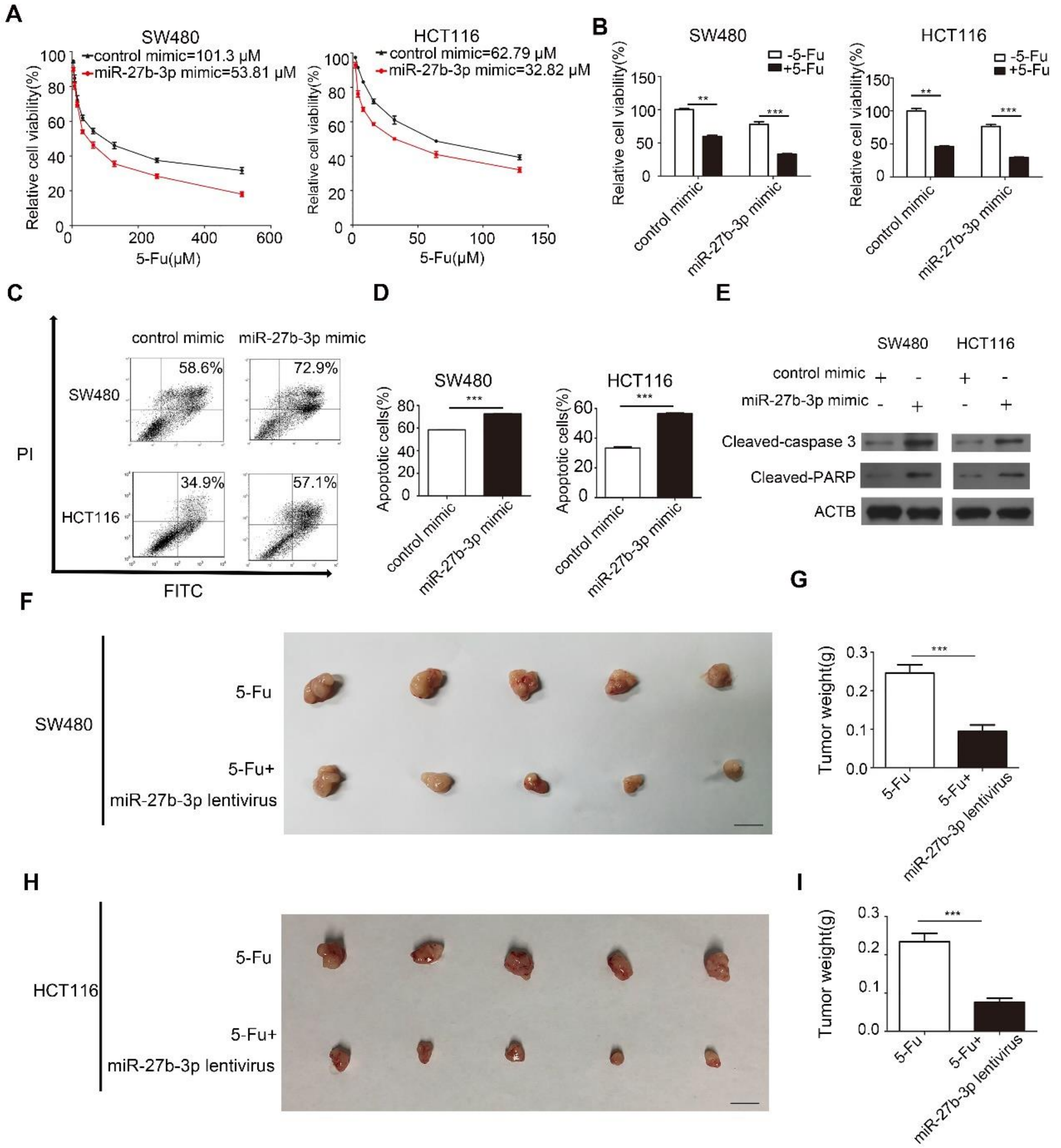

Figure S3. MiR-27b-3p reverses the chemoresistance of CRC cells to 5-Fu. (A) Growth curves of SW480 cells (left) and HCT116 cells (right) after transfection as indicated. (B) The CCK8 assay showed a change in cell viability in response to 5-Fu after transfection of SW480 cells (left) and HCT116 cells (right). (C and D) Cell apoptotic rates of SW480 (upper) and HCT116 (lower) cells were detected by flow cytometry. (C): representative images; (D): quantitative analysis.(E) Cleaved-caspase 3 and PARP expression were observed by western blot in SW480 cells (left) and HCT116 cells(right).(F) Representative images of tumors in nude mice bearing SW480 cells in two groups (n= 5 for each group).

Scale bars: 1 cm. (G) Tumor weights were measured in different groups. (H) Representative images of tumors in nude mice bearing HCT116 cells in different groups (n= 5 for each group). Scale bars: 1 cm. (I) Tumor weights were measured in different groups. **p < 0.01, ***p < 0.001.

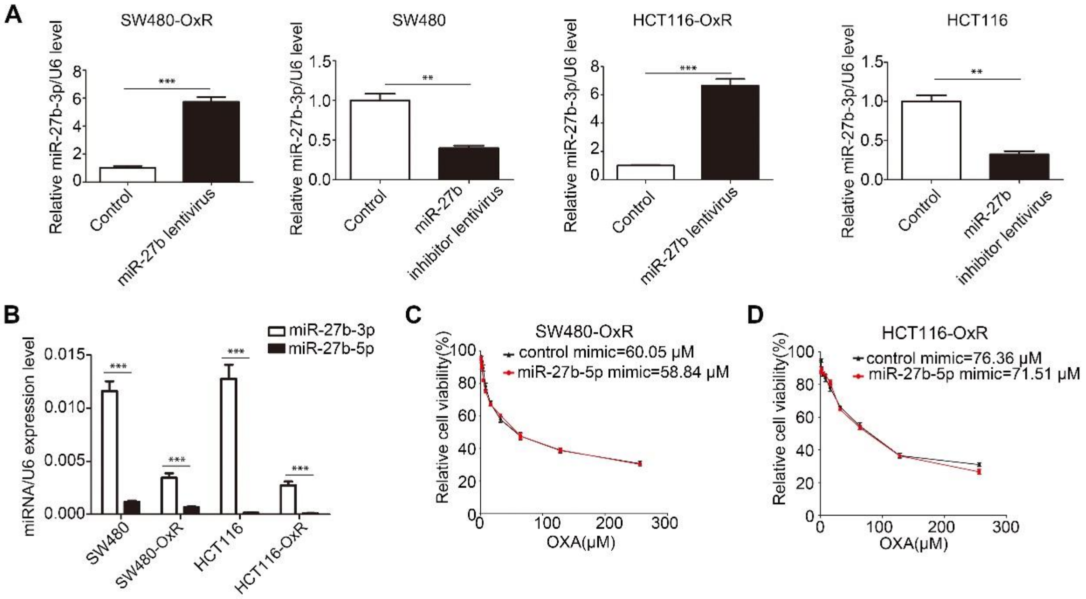

Figure S4. MiR-27b-3p suppresses tumor growth when combined with oxaliplatin in vivo. (A)The expression levels of miR-27b-3p were detected by qRT-PCR in four CRC cells transfected with lentivirus as indicated. (B) The relative levels of miR-27b-5p and miR-27b-3p in SW480, HCT116, SW480-OxR and HCT116-OxR cell lines were determined using qRT-PCR. (C and D) Growth curves of SW480-OxR cells (C) and HCT116-OxR cells (D) after transfection as indicated when treated with different concentrations of 5-Fu. *p < 0.05, **p < 0.01, ***p < 0.001.

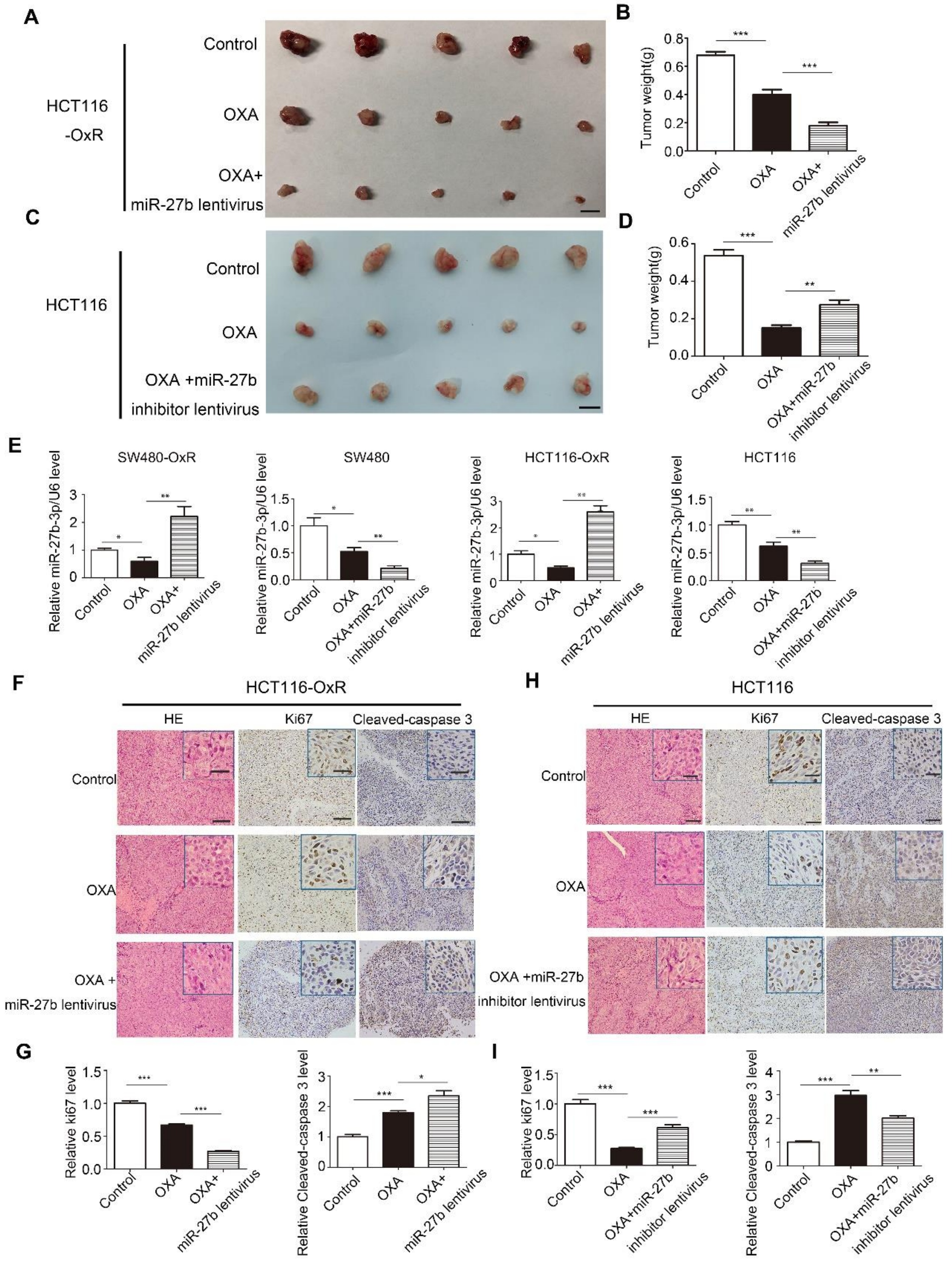

Figure S5. MiR-27b-3p suppresses tumor growth combined with oxaliplatin in vivo. (A) Representative images of tumors in nude mice bearing HCT116-OxR cells in different groups (n= 5 for each group). Scale bars: 1 cm. (B) Tumor weights were measured in different groups. (C) Representative

images of tumors in nude mice bearing HCT116 cells in different groups (n= 5 for each group). Scale bars: 1 cm. (D) Tumor weights were measured in different groups. (E) qRT-PCR detected the expression levels of miR-27b-3p in tumors from mice of each group. (F) Representative images of tumor samples derived from HCT116-OxR group that were stained with H&E (left) and immunohistochemistry of Ki67 (middle) and cleaved-caspase 3 (right). Scale bars: 100 μm; (insets) 25 μm. (G) Statistical analysis of Ki-67 and cleaved-caspase 3 protein levels in (F). (H) Representative images of tumor samples derived from HCT116 group that were stained with H&E (left) and immunohistochemistry of Ki67 (middle) and cleaved-caspase 3 (right). Scale bars: 100 μm; (insets) 25 μm. (I) Statistical analysis of Ki-67 and cleaved-caspase 3 protein levels in (H). *p < 0.05, **p < 0.01, ***p < 0.001.

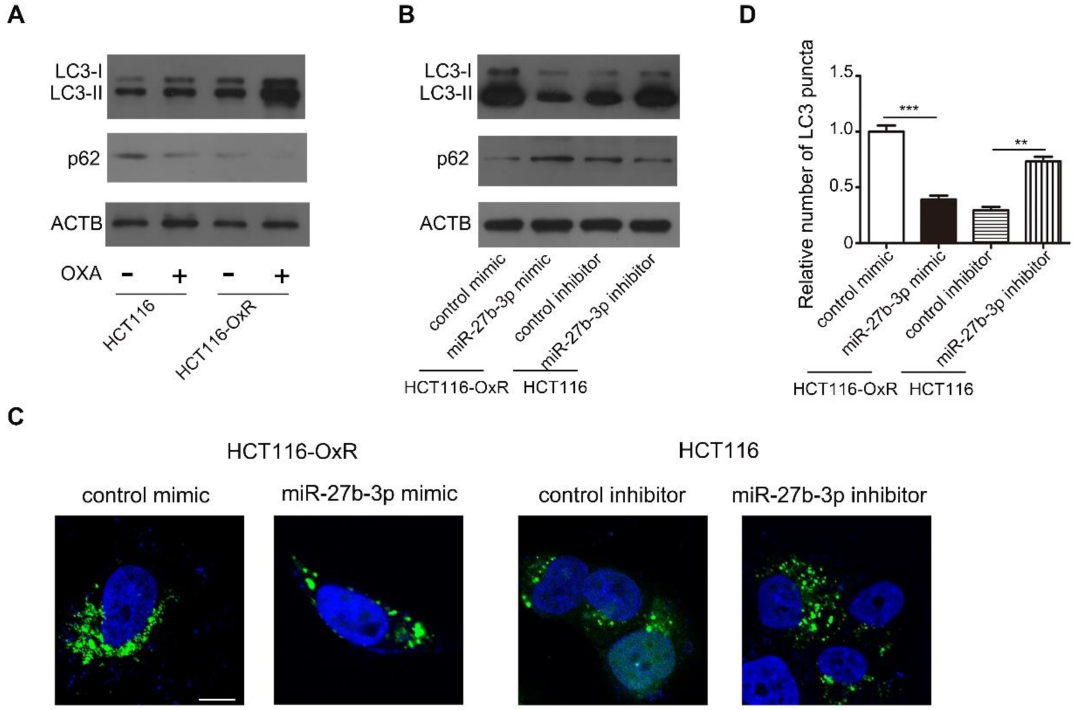

Figure S6. MiR-27b-3p inhibits autophagic activity in chemoresistant CRC cells. . (A) Autophagy element expression levels were detected by western blot in HCT116 and HCT116-OxR cells cultured with oxaliplatin. (B and C) HCT116-OxR and HCT116 cells were transfected with mimics or inhibitor

of miR-27b-3p, respectively. After culturing with oxaliplatin, (B) autophagy element expression levels were detected by western blot, (C) green fluorescent LC3 puncta were observed under confocal microscope, respectively. LC3 puncta per cell were quantified in (D). Scale bar: 10 μm. **p < 0.01, ***p < 0.001.

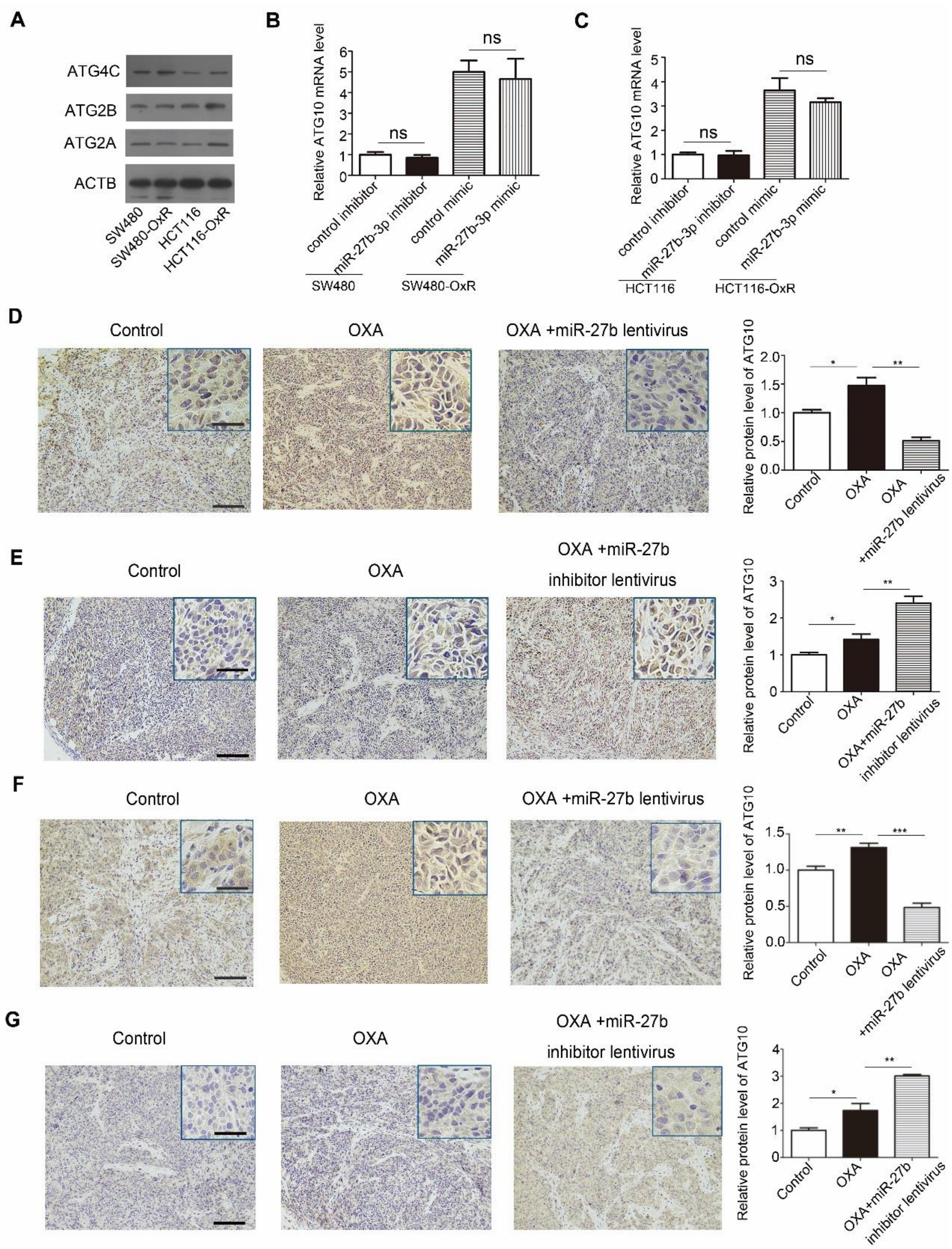

Figure S7. Identification of ATG10 as a direct target of miR-27b-3p. (A) Western blot showing the

expression levels of ATG4C, ATG2A and ATG2B in four CRC cell lines. (B and C) Quantitative analyses of mRNA levels of ATG10 in CRC cells transfected as indicated. (D) Representative images of tumor samples stained for ATG10 by IHC in SW480-OxR group (left). The levels of ATG10 protein expression were measured (right). Bars: (main) 100 μm; (insets) 25 μm. (E) Representative images of tumor samples stained for ATG10 by IHC in SW480 group (left). The levels of ATG10 protein expression were measured (right). Bars: (main) 100 μm; (insets) 25 μm. (F) Representative images of tumor samples stained for ATG10 by IHC in HCT116-OxR group (left). The levels of ATG10 protein expression were measured (right). Bars: (main) 100 μm; (insets) 25 μm. (G) Representative images of tumor samples stained for ATG10 by IHC in HCT116 group (left). The levels of ATG10 protein expression were measured (right). Bars: (main) 100 μm; (insets) 25 μm.*p<0.05, **p<0.01, ***p<0.001.

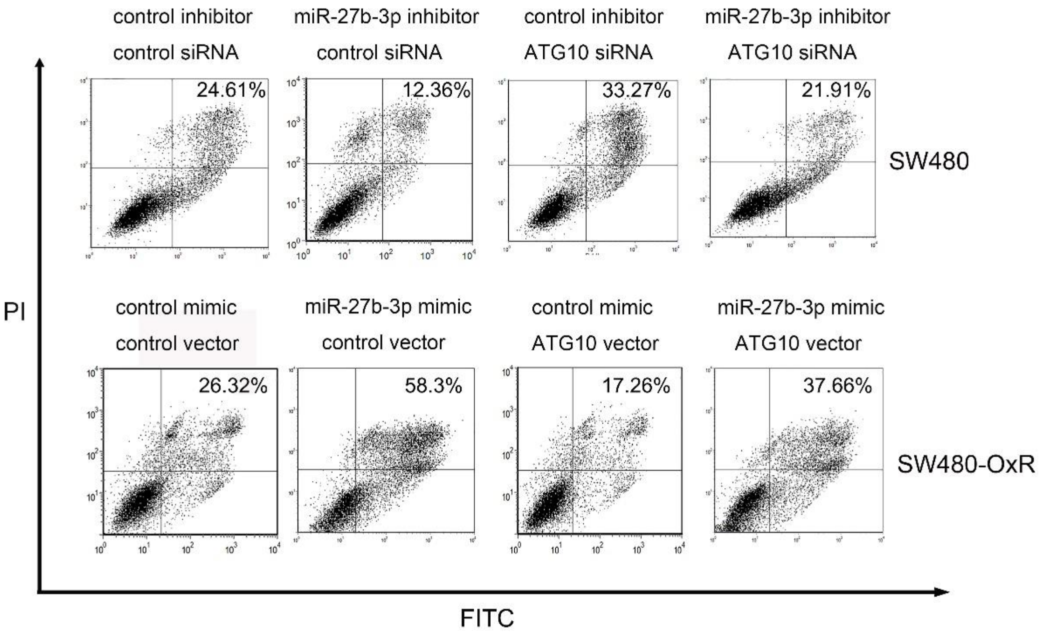

Figure S8. Apoptosis was detected by flow cytometry in SW480 cells and SW480-OxR cells after transfection as indicated, respectively.

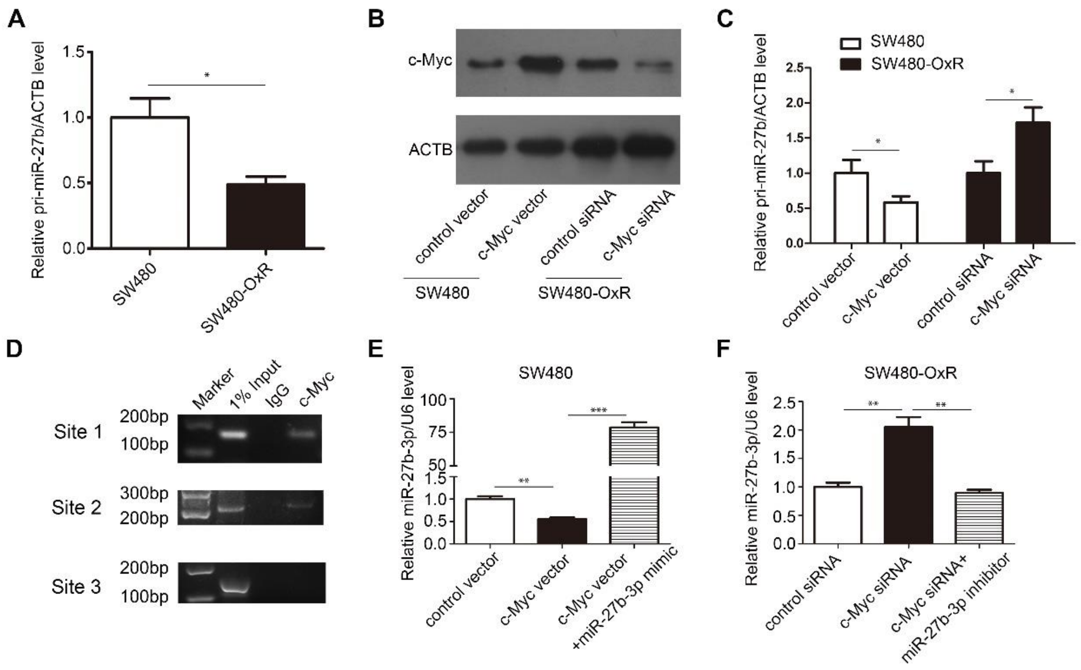

Figure S9. Expression of miR-27b-3p is inhibited by c-Myc. (A) Quantitative analyses of pri-miR-27b levels in SW480 cells and SW480-OxR cells. (B) Western blot analysis of c-Myc protein levels in SW480 cells transfected control vector, c-Myc vector, and SW480-OxR cells transfected with control siRNA and c-Myc siRNA. (C) The influence of c-Myc on modulating the expression levels of pri-miR-27b. (D) ChIP assay for c-Myc occupancy on the miR-27B promoter region. (E and F) MiR-27b-3p expression levels were were detected by qRT-PCR in SW480 cells (E) and SW480-OxR cells (F). *p<0.05, **p<0.01, ***p<0.001

Table S1 Sequences for primers of genes primers and mRNA siRNAs used in this study

Name	Sequence(5'-3')
**siRNA sequences**	
ATG10 siRNA	GGAGUUCAUGAGUGCUAUA
c-Myc siRNA	CGAUGUUGUUUCUGUGGAA
**q-PCR primers**	
ATG10-F	AGACCATCAAAGGACTGTTCTGA
ATG10-R	GGGTAGATGCTCCTAGATGTGA
c-Myc-F	TCAAGAGGTGCCACGTCTCC
c-Myc-R	TCTTGGCAGCAGGATAGTCCTT
ACTB-F	TTCCTTCCTGGGCATGGAGTCC
ACTB-R	TGGCGTACAGGTCTTTGCGG
Pri-miR-27b-F	TTATGCCCAGCGATGACC
Pri-miR-27b-R	GGCTCCAACTTAACTGTCCC
**ChIP primers**	
Site1-F	ACAGAGCACCTGCGGCACA
Site1-R	GGACCTGCCCTCAAGACAC
Site2-F	CACATGTGGCACACACAGTG
Site2-R	ATCTAACAGGTTAATTCCAGGCAC
Site3-F	CTGAAAGAACTCTAATAATGAAAAG
Site3-R	GTTTCGCTACGAGACGCT

Table S3

A miRNA mimics/inhibitors found to sensitize SW480-OxR cells to oxaliplatin in the screening system-

**MiRNA ID**	**%Proliferation (miRNA)**	**%Proliferation,(miRNA +IC_{50} OXA)**
MiRNA mimic		
NC	100	49.7
miR-27b-3p	59.8	20.9
miR-421	67.5	29.6
miR-422a	71.5	34.3
miR-145-5p	73.9	37.1
MiRNA inhibitor		
IN-NC	100	54
miR-10a-5p	67.3	31.6
miR-4734	75.9	38.4
miR-6789-5p	76.1	35.8
miR-4707-5p	87.5	42.6

B miRNA mimics/inhibitors found to sensitize HCT116-OxR cells to oxaliplatin in the screening system-

**MiRNA ID**	**%Proliferation (miRNA)**	**%Proliferation,(miRNA +IC_{50} OXA)**
MiRNA mimic		
NC	100	52.8
miR-27b-3p	63.9	24.5
miR-421	77.9	32.9
miR-422a	77.5	43.2
miR-145-5p	81.5	37.5
MiRNA inhibitor		
IN-NC	100	54
miR-10a-5p	65.9	37.4
miR-4734	68.2	41.2
miR-6789-5p	71.2	39.3
miR-4707-5p	70.8	41.9

Table S4:Clinical information on Cohort 1 (fresh tissues)					
**Patients**	**Gender**	**Age(years old)**	**TNM stage**	**Tumor size(cm)**	**Cancer subtype**
1	Female	76	IIB	4	colon carcinoma
2	Male	50	IIB	6.5	colon carcinoma
3	Male	51	IIIA	5	rectal carcinoma
4	Male	61	IIB	3.5	colon carcinoma
5	Female	47	IIA	3	rectal carcinoma
6	Female	62	IIA	4	rectal carcinoma
7	Female	70	IIA	7	colon carcinoma
8	Male	27	IIIA	5	colon carcinoma
9	Male	50	IIIB	6	colon carcinoma
10	Male	55	IIB	8	colon carcinoma
11	Male	55	IIIA	8	colon carcinoma
12	Male	61	IIB	4	colon carcinoma
13	Male	64	IIB	9	colon carcinoma
14	Female	64	IIA	2	colon carcinoma
15	Male	65	IIB	3.4	rectal carcinoma
16	Female	65	IIB	3.5	rectal carcinoma
17	Male	64	IIIA	4	colon carcinoma
18	female	63	IIB	2.5	colon carcinoma
19	Male	48	IIB	1	colon carcinoma
20	Male	66	III	3.5	colon carcinoma

Table S5:Clinical information on Cohort 2 (paraffin tissues)								
**Patients**	**Gender**	**Age(years)**	**Tumor size(cm)**	**Histological Grade**	**TNM stage**	**Recurrence**	**FolLow-up time (months)**	**Cancer subtype**
1	Male	54	2.5	II	IIB	NO	60	rectal carcinoma
2	Female	54	4.5	II	IIB	NO	49	rectal carcinoma
3	Male	49	2	II	IIA	NO	60	rectal carcinoma
4	Male	70	9	II	IIIC	YES	30	colon carcinoma
5	Male	66	4.5	II	IIB	NO	37	rectal carcinoma
6	Female	77	6.5	II	IIB	NO	62	colon carcinoma
7	Male	72	5	II	IIB	NO	59	rectal carcinoma
8	Female	58	5	II	IIIB	NO	62	rectal carcinoma
9	Male	39	4	II	IIIB	YES	26	rectal carcinoma
10	Male	46	4.5	II	IIA	NO	51	rectal carcinoma
11	Male	75	3	II	IIA	NO	50	colon carcinoma
12	Male	50	2.5	II	IIB	NO	60	rectal carcinoma
13	Male	71	3.5	II	IIIC	YES	28	colon carcinoma
14	Female	55	5	I	IIB	NO	61	rectal carcinoma
15	Male	61	7	II	IIIB	YES	16	colon carcinoma
16	Male	63	7	II	IIIB	NO	38	rectal carcinoma
17	Male	66	5	II	IIIB	YES	18	rectal carcinoma
18	Female	63	7	II	IIB	NO	60	colon carcinoma
19	Female	48	5	II	IIB	NO	60	rectal carcinoma
20	Male	66	6	III	III	NO	61	colon carcinoma
21	Male	82	6.5	II	IIB	NO	60	rectal carcinoma
22	Female	81	6.5	II	IIA	NO	60	rectal carcinoma
23	Male	65	3.5	III	IIA	YES	13	rectal carcinoma
24	Female	61	4.5	II	IIB	NO	62	colon carcinoma
25	Male	56	3	I	IIIB	NO	57	rectal carcinoma
26	Male	56	3	II	IIB	NO	61	rectal carcinoma
27	Female	77	6	II	IIB	NO	62	colon carcinoma
28	Male	42	3	II	IIB	NO	55	rectal carcinoma
29	Male	55	4	II	IIIB	NO	60	rectal carcinoma
30	Male	37	4	II	IIB	NO	60	colon carcinoma
31	Female	83	3	II	IIIB	YES	17	colon carcinoma
32	Female	47	5	II	IIIB	NO	62	rectal carcinoma
33	Male	37	4	I	IIIB	YES	22	colon carcinoma
34	Male	56	5	II	IIB	YES	17	colon carcinoma
35	Female	71	7	III	IIA	NO	61	colon carcinoma
36	Male	53	8	III	IIIB	YES	10	colon carcinoma
37	Female	48	4.5	II	IIIB	NO	62	colon carcinoma
38	Male	44	4.5	II	IIB	NO	60	colon carcinoma
39	Male	43	5	III	IIIB	YES	36	rectal carcinoma
40	Male	62	6	I	IIIA	NO	62	colon carcinoma
41	Female	63	7	II	IIIB	YES	14	colon carcinoma
42	Male	61	7	II	IIA	NO	57	rectal carcinoma
43	Male	65	13	II	III	NO	60	rectal carcinoma
44	Female	52	4	II	IIIB	YES	9	rectal carcinoma
45	Female	60	4	II	IIIB	YES	9	rectal carcinoma
46	Male	41	5	I	IIB	NO	60	colon carcinoma
47	Male	57	3	II	IIIC	NO	60	rectal carcinoma
48	Male	84	6.5	II	IIIC	YES	9	colon carcinoma
49	Male	48	2	I	IIIA	NO	62	colon carcinoma
50	Male	48	5	II	IIA	NO	60	colon carcinoma
51	Female	62	4	III	IIIC	YES	3	rectal carcinoma
52	Male	68	5	II	IIB	NO	60	rectal carcinoma
53	Female	85	7	II	IIIB	YES	10	rectal carcinoma
54	Male	31	2.5	II	IIB	NO	60	colon carcinoma
55	Female	62	3	II	IIB	NO	60	rectal carcinoma
56	Male	64	4.5	II	IIB	YES	9	rectal carcinoma
57	Male	51	7	II	IIB	NO	29	colon carcinoma
58	Female	27	9	II	IIIB	YES	17	colon carcinoma
59	Female	70	2.5	II	IIB	YES	6	rectal carcinoma
60	Female	60	4	II	IIB	YES	15	rectal carcinoma
61	Female	60	5	II	IIIB	YES	10	rectal carcinoma
62	Male	55	8	II	IIIC	YES	3	colon carcinoma

Table S6 Correlation of miR-27b-3p expression in colorectal cancer tissue of cohort 2 (62 cases) with patients' clinicopathological variables

Variables	miR-27b-3p expression			*P*-value
	All cases n=62	Low expression n = 34	High expression n = 28	
**Gender**				
Male	39	20	19	0.46
Female	23	14	9	
**Age (years)**				
≤ 58.6	30	18	12	0.429
> 58.6	32	16	16	
**Tumor size (cm)**				
≤ 5	42	23	19	0.986
> 5	20	11	9	
**Grade of differentiation**				
I	6	4	2	0.657
II	50	26	24	
III	6	4	2	
**TNM stage**				
II	34	16	18	0.175
III	28	18	10	
**Relapse**				
No	39	17	22	0.021
Yes	23	17	6	

1. Statistical comparison was performed by using x^2 test in SPSS 20.0.

2. Analysis was conducted on 62 cases shown in Supplementary Table S5.
